# From nutrients to competition processes: Habitat specific threats to *Arnica montana* L. populations in Hesse, Germany

**DOI:** 10.1371/journal.pone.0233709

**Published:** 2020-05-29

**Authors:** Verena Hollmann, Tobias W. Donath, Florian Grammel, Tina Himmighofen, Ute Zerahn, Ilona Leyer

**Affiliations:** 1 Department of Applied Ecology, Hochschule Geisenheim University, Geisenheim, Germany; 2 Department of Landscape Ecology, Institute for Natural Resource Conservation, Kiel University, Kiel, Germany; 3 Institute of Landscape Ecology and Resource Management, Interdisciplinary Research Centre, Justus Liebig University Giessen, Gießen, Germany; Estacion Experimental de Zonas Aridas, SPAIN

## Abstract

Populations of *Arnica montana*, a characteristic species of nutrient poor grasslands in Central Europe, have been deteriorating over the last decades, especially in lowland regions. Population size has been declining and signs of sexual reproduction are scarce. To start a long-term regeneration program, we investigated the major habitat specific drivers for the decline in Hesse, Germany. Firstly, we conducted a field study to analyze habitat characteristics of 32 Hessian lowland sites, comparing those on which this species has become extinct during the last 15 years with sites of small and declining, as well as large, stable populations. We compared habitat traits focusing on soil parameters, nutrients, and vegetation characteristics. Secondly, we set up a greenhouse experiment to study the response of *A*. *montana* seedlings to competition and nutrient input to assess the effects of competition pressure and fertilization. The results show lower carbon-to-nitrogen ratios and higher Ellenberg nitrogen indicator values on sites with extinct populations compared to existing populations. Both pH and Ellenberg soil reaction indicator values were higher on sites with extinct populations. In the greenhouse, the combination of nitrogen addition and competition resulted in lower seedling numbers. While rosette size was not dependent on fertilization, growth was strongly enhanced in the plots lacking vegetation. Both studies suggest that soil nutrient enrichment followed by competition pressure diminishes the number of safe sites for *A*. *montana* seedling recruitment and establishment and negatively impacts the growth of existing rosettes, thus leading to the continuous decline of populations. There is an urgent need for actions to reduce unintentional nitrogen deposition in the remaining nutrient poor areas as well as to modify land use to withdraw nutrients from enriched soils in order to preserve the remaining *A*. *montana* populations and to create bare ground for the safekeeping and enhancement of self-sustainable populations.

## Introduction

Nutrient poor, acidic grass- and heathlands contribute significantly to the biodiversity in Central European ecosystems but have been declining for decades, both in quality and area [1]. While the strong decline of these plant communities is well documented [2], this process does not necessarily translate into the decline of typical species (e.g. *Calluna vulgaris*, *Carex pilulifera*, *Danthonia decumbens*, *Luzula campestris*) or lead to them becoming red-listed [3]. The main reason for the latter phenomenon is that these species also occur in other nutrient poor habitats [4] and are therefore not regarded endangered across habitat types [2, 3]. The opposite is true for the medicinal plant *Arnica montana* L., whose occurrence is much more confined to acidic, nutrient poor sites [5]. The species is spread across Europe from the Pyrenees to the Carpathians Mountains and Ukraine with the Alps being the southern distribution border and Southern Scandinavia being the northern outermost edge [5, 6]. Its main distribution area lies in Germany´s (sub-)mountain ranges making it a species of national responsibility [5, 7]. Although protected by national and international law, population density and size has been declining strongly over the last decades.

Miscellaneous causes for this decline have been recognized in Germany and neighboring countries including habitat loss, habitat fragmentation, habitat isolation, unsuitable management techniques, soil acidification, illegal collection of flower heads as well as the effects of herbivores and both invasive and aggressively spreading native plant species, e.g. *Lupinus polyphyllus* and *Pteridium aquilinum* [8–11]. One of the most investigated possible causes is nutrient, especially nitrogen (N), excess [12–14]. There are many reports on how grassland vegetation can respond to varying N input. Decreased light availability and lower density in terms of gap dynamics, both caused by the proliferation of strong competitors as the result of increased nutrient levels, are key processes that lead to declining species diversity in these systems [15–17]. Changes in grassland composition (e.g. the transformation of heathlands into grasslands) and subsequent changes in the pressures of competition can be linked to just slightly elevated N deposition rates [18] and might offer a major explanation for the unfavorable state of many *A*. *montana* populations [13, 14].

Still, studies have drawn different conclusions regarding the ultimate threat or combination of threats to *A*. *montana* [8–14]. The ambiguity of these results suggests that specific threats depend on the location and might not be transferable across the whole distribution range. Therefore, in order to set up a long-term regeneration program for Hessian *A*. *montana* populations, knowledge about the particular threats is needed as a basis for the development and implementation of precise countermeasures and management regimes.

In Hesse, mountainous populations appear relatively stable while the decrease is much more noticeable in lowland regions (below 500 m above sea level) [11, 19], although a considerable part of these populations is situated in nature reserves and other protected areas where stricter nature conservation management is applied. About 60–70 lowland populations are currently present with the majority being remnants of formerly large *A*. *montana* populations with a low number of rosettes and only few or no inflorescences today. Regarding the sexual reproduction, populations of *A*. *montana* in Hesse face therefore inauspicious prospects as no seedlings have been spotted on most Hessian lowland sites in recent years [11]. The species can, however, spread clonally. It is well known, however, that extensive clonal growth can have negative effects on sexual propagation, e.g. by the absence of mating groups required for outcrossing in large clonal clusters [20, 21]. Sexual reproduction, furthermore, is of utmost importance for plant species for maintaining genetic diversity and gene flow responses in the landscape [22]. Colling et al. [23] demonstrated this was the case for *Scorzonera humilis* with lack of recruitment, indicated by low numbers of seedlings, being among the main causes for the decline of this rare species.

The purpose of our study was to identify the crucial habitat related drivers for the decline of *A*. *montana* in Hesse in general and specifically for the lack of seedlings and juvenile plants, as well as to determine environmental variables that indicate the risk potential for populations. With this aim, a field study and a greenhouse experiment were conducted. Firstly, we analyzed habitat characteristics of 32 sites in Hesse, comparing those on which the species has become extinct during the last 15 years with sites of small, declining, as well as large, stable populations of *A*. *montana*. Topographic, soil, and vegetation parameters were measured and related to the different site categories. Secondly, we asked if the bad germination rates and establishment success of *A*. *montana* in the field could be verified and attributed to the aforementioned environmental changes in nutrient availability and increased effects of competition. Thus, we conducted a greenhouse experiment analyzing the effects of fertilization and competition pressure on germination and establishment of *A*. *montana* seedlings.

Specifically, we addressed the following research questions: (i) What are crucial habitat specific features associated with the decline of *A*. *montana* in Hesse? (ii) Are germination rates and seedling performance affected by interspecific competition as well as nutrient input?

## Material and methods

### Study species

*A*. *montana* (Asteraceae) is a perennial, herbaceous plant and a characteristic species of nutrient poor grass- and heathlands (*Nardetalia*, *Calluno-Ulicetalia*) [24, 25]. This calcifuge and heliophytic species is pollinated by a variety of insect species and its plumed achenes are dispersed by wind though its heavy seeds do not spread across long distances [26]. *A*. *montana* seeds do not require stratification and despite being able to germinate in the dark, low light availability (e.g. in dense vegetation) prevents seedlings from developing further [27].

### Study sites and sampling

**Fig 1:**
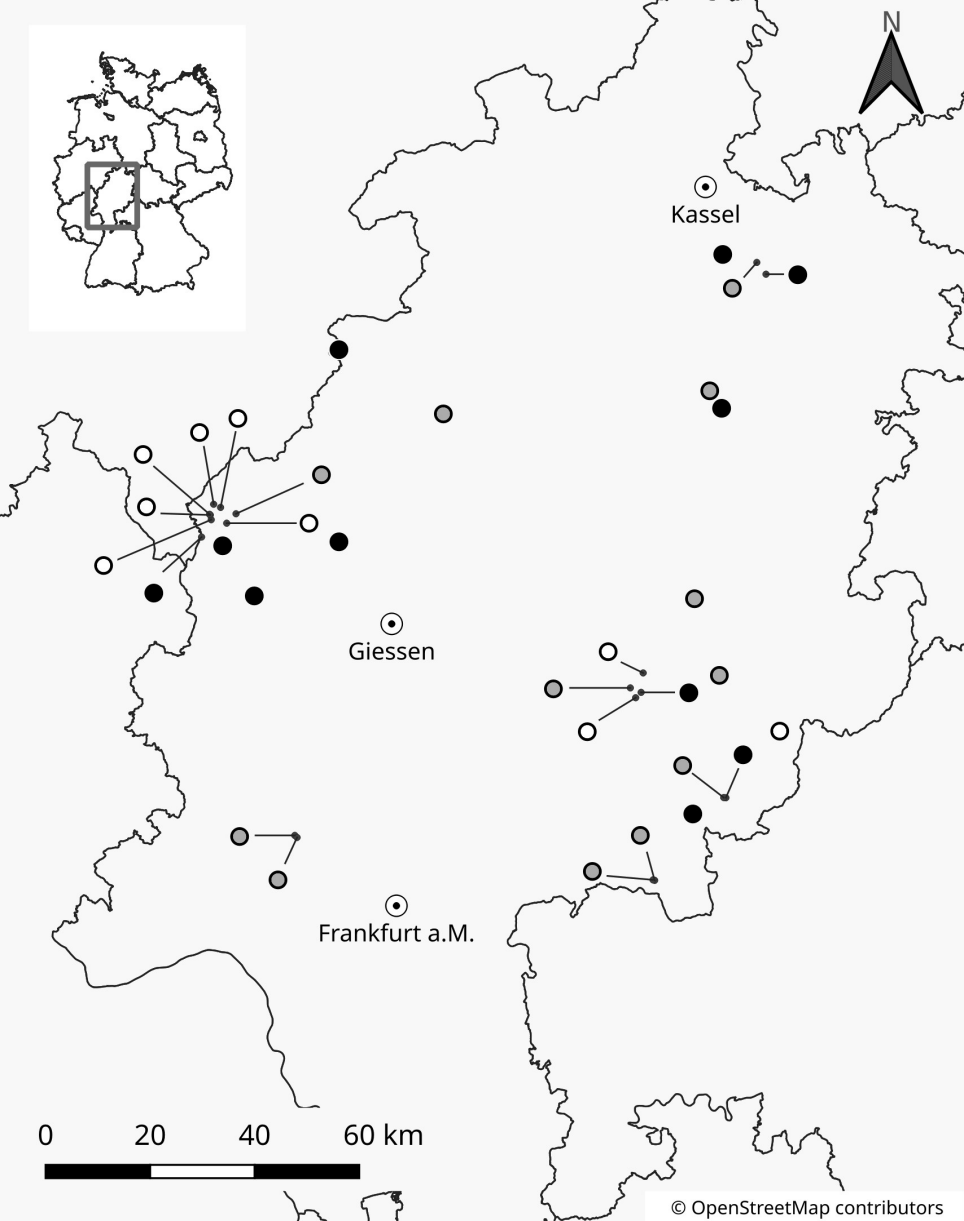
**Location of the 32 field sites of extinct (white), small (gray) and large (black) *A*. *montana* populations.** All sites were located in Hesse, Germany.

We compared topographic, soil and vegetation parameters among three classes of lowland sites (below 500 m a.s.l.): extinct *A*. *montana* populations (verifiably undergoing extinction during the last 15 years; no rosettes present; 9 sites), small (1–400 rosettes per site; 12 sites), and large populations (more than 400 rosettes per site; 11 sites). All sites were located in Hesse, Germany (Fig 1), representing about 40% of known lowland populations of *A*. *montana* in the study region. Among the populated sites, the rosette numbers ranged from 11 to 8875. As it was not possible to differentiate between clones on sight, the rosette numbers were not equivalent to the numbers of genetic individuals. Most measurements were taken in the summer of 2013 with the exception of biomass samples for nutrient analysis which were collected in 2015. Altitude above sea-level, slope, and other main characteristics were assessed for all sites (S1 Table). Hessen-Forst FENA granted permission to collect plant samples and enter nature reserves (reference number IV.2 R 28).

### Soil measurements

Five soil samples (depth 0–10 cm) for each site were taken directly around existing *A*. *montana* clusters. At extinct population sites, either the area around a formerly documented *A*. *montana* patch or an area that seemed most suitable for the species was selected. Samples were pooled per site, dried at 40°C and sieved to retain particles above 2 mm. All measurements were conducted according to the standard guidelines issued by the association of German agricultural analytic and research institutes [28]. The samples were analyzed for pH, soil type, plant available phosphorus (P), potassium (K), magnesium (Mg), and carbon-to-nitrogen ratio (C:N).

pH was measured three hours after applying a 0.01 M calcium-chloride solution while soil type was determined using a texture triangle. Silt and clay were separated via sediment analysis and the sand portion was isolated by wet sieving. For the measurement of plant available P and K, the soil samples were blended with calcium-acetate-lactate extract. Afterwards, P was measured using a photometer and staining with ammonium-molybdate, while K content was determined via a flame photometer. To quantify plant available Mg, soil was mixed with a calcium-chloride solution and its suspension measured in an atomic absorption spectrometer. Soil C and N were determined using an elemental analyzer by means of combustion and following gas chromatography in the thermal conductivity detector. The C:N ratio was calculated, and organic matter content determined by multiplying the C content with the factor 1.724 [29, 30].

### Vegetation-related measurements

Vascular plant species were counted on two plots of 1 m^2^ per site using a 1 m^2^ grid divided into 25 equally-sized cells of 20 cm x 20 cm. The grid was placed on *A*. *montana* rosette free ground as close as possible to the largest *A*. *montana* group to represent conditions most suitable for the species (max. distance of 0.5–3 m). At extinct population sites, the grid was either positioned near a formerly documented *A*. *montana* group or on a vegetation patch that seemed most typical for this species. Plot recordings consisted of presence-absence data of species in each grid cell. For the analyses, the number of presences of each species in the grid cells of both plots per site was used (summing up to a maximum of 50) and the number of species was calculated for each site. Proportion of bare soil on 1 m^2^ of ground was estimated. Moreover, above-ground biomass on an area of 32 cm x 32 cm was retrieved, sorted into vascular plant biomass, moss and litter, then dried, weighted, and results extrapolated to 1 m^2^. The location of the biomass samples was chosen according to the procedure of placing the vegetation grids mentioned above.

In the summer of 2015, samples of *A*. *montana* and of the surrounding biomass were collected. Ten leaves of *A*. *montana* rosettes were gathered per site. For the surrounding biomass, the largest group of *A*. *montana* rosettes was selected and a grid of 10 cm x 10 cm was placed on a representative area from which all above-ground biomass was collected. This procedure was repeated three times around the mentioned *A*. *montana* group and the four samples were pooled for this site. At extinct population sites, either the area around a formerly documented *A*. *montana* patch, or an area that seemed most suitable for the species, was selected and samples were collected as aforementioned. The samples were dried, ground, and analyzed regarding macro- as well as micronutrients in the leaves (N, P, K, Ca, Mg, Fe, Zn, Mn, and Cu) and were prepared according to the procedures of wet combustion and Kjeldahl digestion [31] and measured using a Foss Tecator FIAstar 5000 Analyzer.

Ellenberg indicator values are based on the ecological requirements of a plant species towards a specific site factor and are usually expressed as numbers from 1 to 9 [32]. They are derived empirically from field studies and widely used [33]. It is important to note that the indicator values do not reflect the optimal conditions for a species in monoculture but rather its peak occurrence and habitat niche under environmental field conditions and interspecific competition [34]. Species composition of plots thus reflect integrated information about the conditions pertaining at a site over time without the need to measure all possible variables.

As for the analysis of the vegetation data, mean Ellenberg indicator values for moisture (F), reaction (R), and nitrogen (N) were calculated for each site [32]. Reaction value (R) assesses the occurrence of a species depending on soil acidity and is not equivalent to pH. Only presence-absence data of species abundance were used to compute mean indicator values (i.e. resulting in unweighted means) as to avoid overestimation of rampantly growing species [33]. Species without existing indicator values were excluded from the analyses.

### Greenhouse experiment

To test the effects and interactions of fertilization and interspecific competition on the germination and growth of *A*. *montana*, a greenhouse experiment was conducted from October 2013 to March 2014. A full-factorial design was determined using three competition levels (no, moderate, and high competition) and two fertilization levels (without and with fertilizer).

The competition levels were simulated using a seed mixture of three grass species commonly found alongside *A*. *montana*: *Agrostis capillaris*, *Festuca rubra*, and *Anthoxanthum odoratum*. The moderate competition level included 5 ml of the seed mixture while 20 ml of the mix was used for the high competition treatment. Each 5 ml seed mix contained approximately 4000 *A*. *capillaris*, 400 *F*. *rubra* and 450 *A*. *odoratum* seeds with the 20 ml mixes containing four times the counts. At the start of the experiment, boxes (45 cm x 29 cm x 6 cm) were filled with soil from an acidic, nutrient depleted grassland. Large rocks, vegetation, and root residues had been removed from the soil beforehand. The grass seed mixture was spread onto the soil according to the competition treatment levels (n = 10 per treatment) and after five weeks half of the boxes of each treatment were fertilized using compound fertilizer (Manna Lin F, NPK ratio of 8:8:6). The amount of N in the applied solution corresponded to 12 kg/ha. Controls were provided with an equal amount of water at the time of fertilization.

After seven weeks of growth, the grasses were cut to a height of 10 cm and weeds were removed from all boxes. We then planted *A*. *montana* seeds that had been collected in the previous summer from two large populations in the Lahn-Dill-Kreis, Hesse, Germany. In each box, we sowed 20 *A*. *montana* seeds placing them onto the soil using pincers. After 90 days, half of the boxes were fertilized again.

Boxes were watered with rainwater throughout the experiment and kept under constant temperature and photoperiod (15/18°C and 10:14 h dark/light). To account for heterogeneous conditions in the greenhouse, boxes were shuffled on a weekly basis. Three response variables were measured after 90 and 150 days following the seeding of *A*. *montana*: (i) number of seedlings in each box, (ii) height, and (iii) width of all rosettes.

### Statistical analysis

Variables taken from the field measurements were tested for differences between population size categories using analysis of variance (ANOVA) with following post-hoc Tukey´s HSD test for corrected pairwise comparisons. Lilliefors test for normality and Brown-Forsythe test for homogeneity of variances was used beforehand. Moreover, a visual assessment of the quantiles of the model´s residuals was conducted. Where requirements of normality and homogeneity of variances were not met, data were transformed or non-parametric Kruskal-Wallis one-way analysis of variance and post-hoc Kruskal-Wallis test was conducted (see S2 Table for details). Correlations of all parameters (excluding species composition data) were conducted for the subsequent elimination of strongly correlating variables in later analyses and for the examination of potential, unanticipated relationships. Pearson´s product-moment correlation coefficient, corrected for multiple comparisons, and Spearman´s rank correlation coefficient as a non-parametric measure test were used for these correlations.

Principal component analysis (PCA) was performed using the topographic, soil, and vegetation parameters. For this analysis, strongly correlating variables were eliminated (silt and clay content, C and N content, species number) and nutrient measurements of the surrounding biomass and *A*. *montana* leaves were excluded as they were not sampled during the same time frame. All parameters were standardized to zero mean and unit variance prior to the PCA. A biplot graph was computed displaying the scores of population sites and loadings of parameters.

To analyze variation in species composition in relation to population size categories, a preliminary detrended correspondence analysis (DCA) was performed. To assess the suitability of the chosen method, we considered the length of gradient which is a measure for the species turnover [35, 36]. The lengths of gradient of 3.3 (1^st^ axis) and 2.1 (2^nd^ axis) indicated that linear models were more appropriate than unimodal ones and therefore a PCA was conducted on the species data [36] which were Hellinger-transformed [37]. Species with less than three occurrences were left out from the analysis reducing the number of species from 112 to 66 in total. To be able to interpret the analysis with external variables, abiotic and biotic variables which were significantly related to the PCA axes (< 0.05, based on a permutation test with 10,000 permutations) were included post-hoc by projection.

For the greenhouse experiment, analysis of variance was carried out to test for significant differences between competition levels and fertilization regarding germination and seedling performance of *A*. *montana* seeds, followed by the above-mentioned post-hoc tests. All statistical analyses were conducted using the software R 3.6.1 [38] and specifically the packages ggplot2 (version 3.2.1) [39] and Vegan (version 2.5–6, e.g. prcomp, ordiplot, envfit) [40].

## Results

### Site characteristics

**Fig 2:**
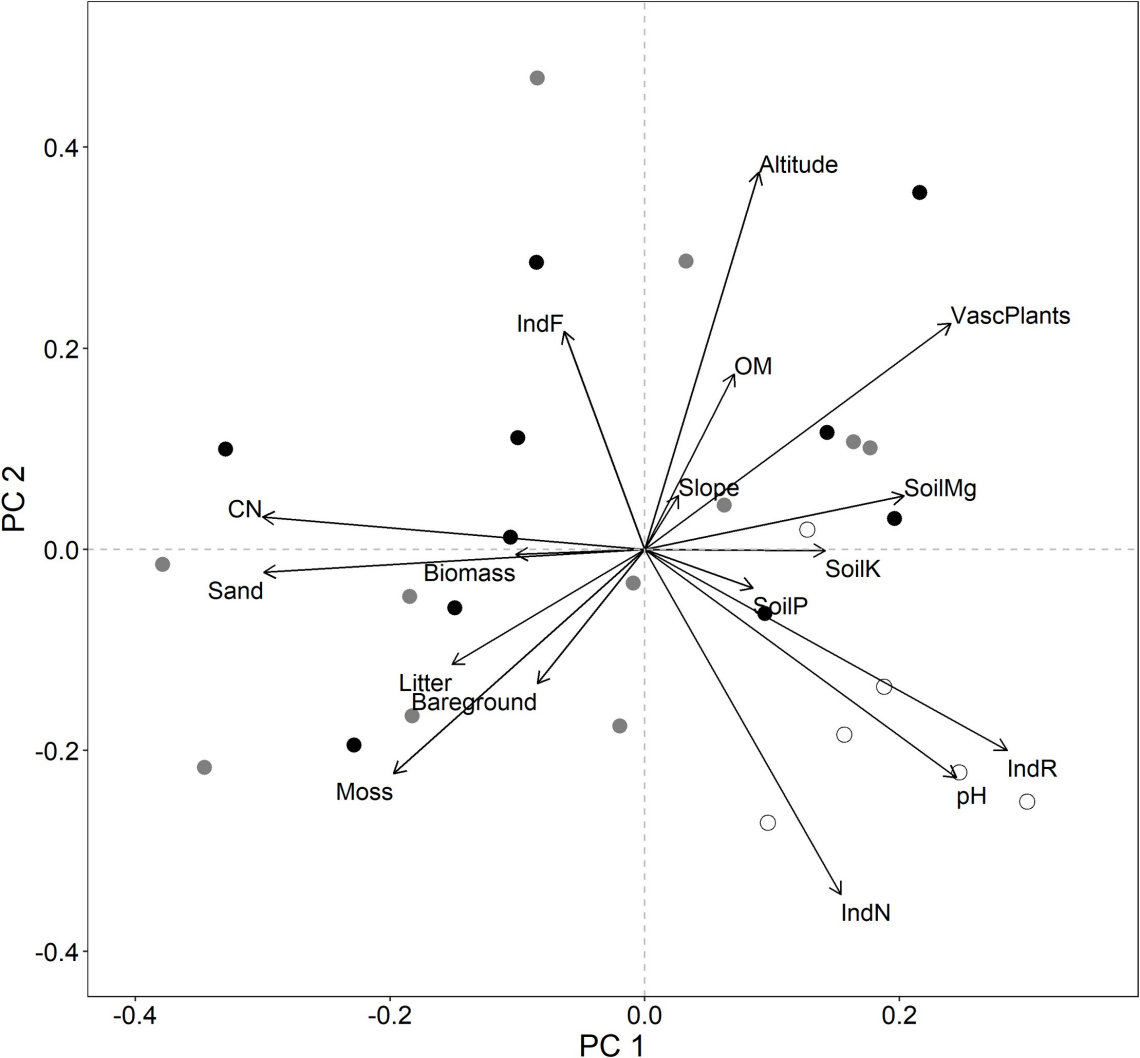
Biplot showing results of the PCA for abiotic and biotic variables from the field study. Topographic, soil, and vegetation parameters are shown as projection arrows onto the principal components. Extinct (white), small (gray) and large (black) *A*. *montana* population sites are displayed as objects. The first two principal components are displayed with 39.5% of variance explained (24.6%, 14.9%). As to present a complete overview of the data set, all variables remaining after removing highly correlated ones are shown; though not necessarily discussed in the text. The following abbreviations are used in the figure: OM–organic matter, CN–soil C:N ratio, IndR, IndF, IndN–Ellenberg indicator values for reaction, moisture and nitrogen; VascPlants–vascular plant biomass on 1 m^2^.

Principal component analysis on environmental parameters showed that extinct population sites clustered regarding the first two principal components while small and large populations were more widespread and not distinctly grouped (Fig 2). Sites without *A*. *montana* were associated with higher values of pH and Ellenberg indicator R and N values whereas they showed negative relationships with sand content, indicator F, and soil C:N ratio.

### Soil characteristics

Grain size distribution was not significantly different between sites of extinct, small, and large *A*. *montana* populations (χ^2^ = 4.36, p = 0.11 for sand; F_2,29_ = 2.59, p = 0.09 for silt), although extinct populations showed lower sand, and higher silt content, compared to still existing populations (with no differences between values of small and large populations, S1 Fig). Clay content was similar for all size categories (S1 Fig).

**Fig 3:**
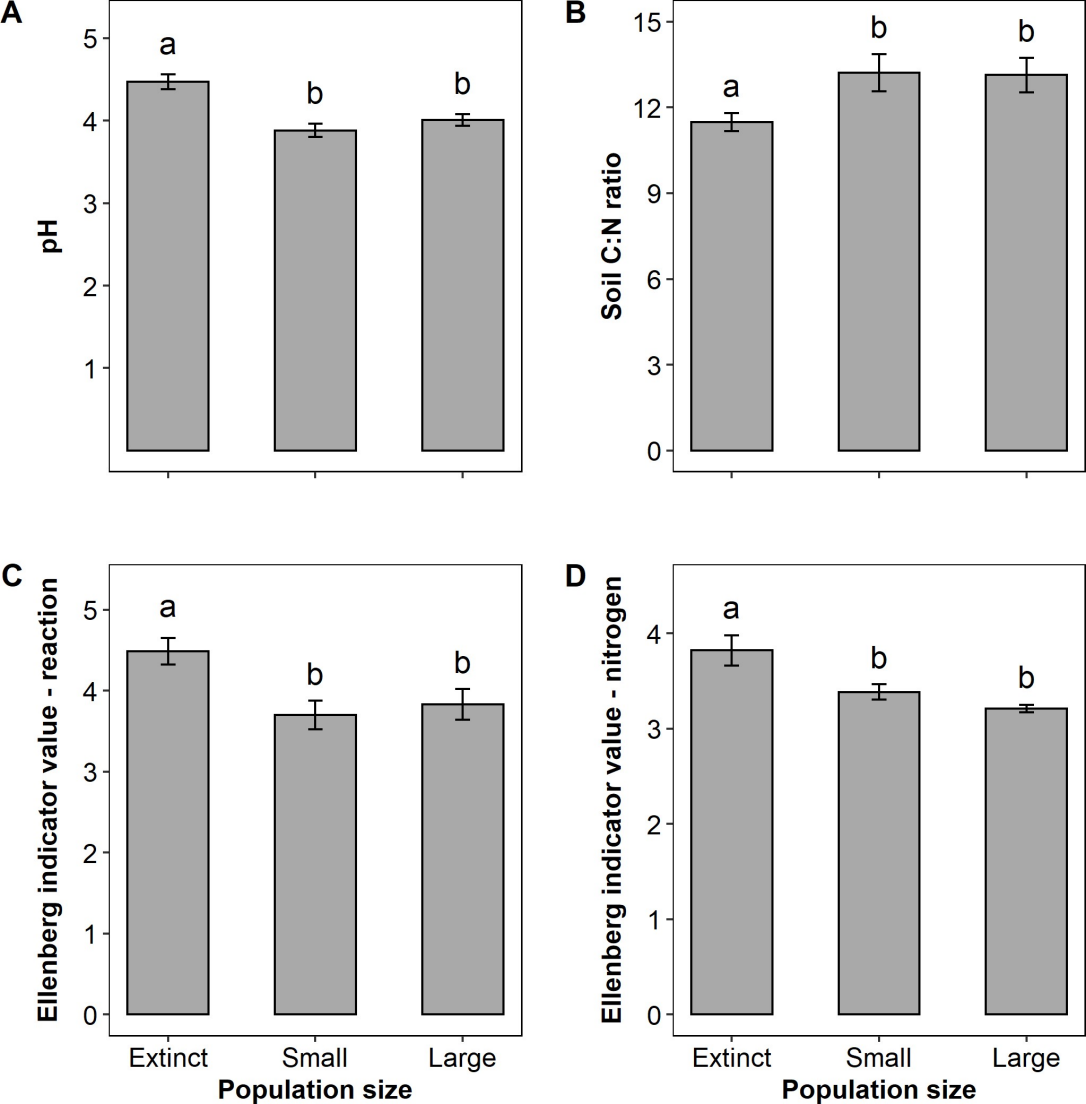
Mean site characteristics for the three population size categories (± SE). Shown are pH (A), soil carbon-to-nitrogen ratio (B), Ellenberg indicator value for reaction (C) and for nitrogen (D). Different letters indicate significant differences between population size categories (p < 0.05).

pH was significantly higher on extinct plant population sites compared to sites with existing populations (Fig 3A, F_2,29_ = 13.32, p < 0.001). Extinct population sites exhibited a significantly lower C:N ratio as opposed to sites with *A*. *montana*, independent of population size (Fig 3B, χ^2^ = 7.24, p = 0.027).

Soil nutrients (P, K, Mg) did not reveal specific findings (S2 Table). Soil P did not differ between population size categories (F_2,29_ = 1.92, p = 0.17) whereas soil K and Mg were significantly different (F_2,29_ = 4.80, p = 0.017 for K, F_2,29_ = 6.05, p = 0.006 for Mg). Soil K and Mg were both lowest on small population sites. Soil K was highest on extinct population sites, while soil Mg was highest on large *A*. *montana* population sites.

### Vegetation composition and biomass measurements

**Fig 4:**
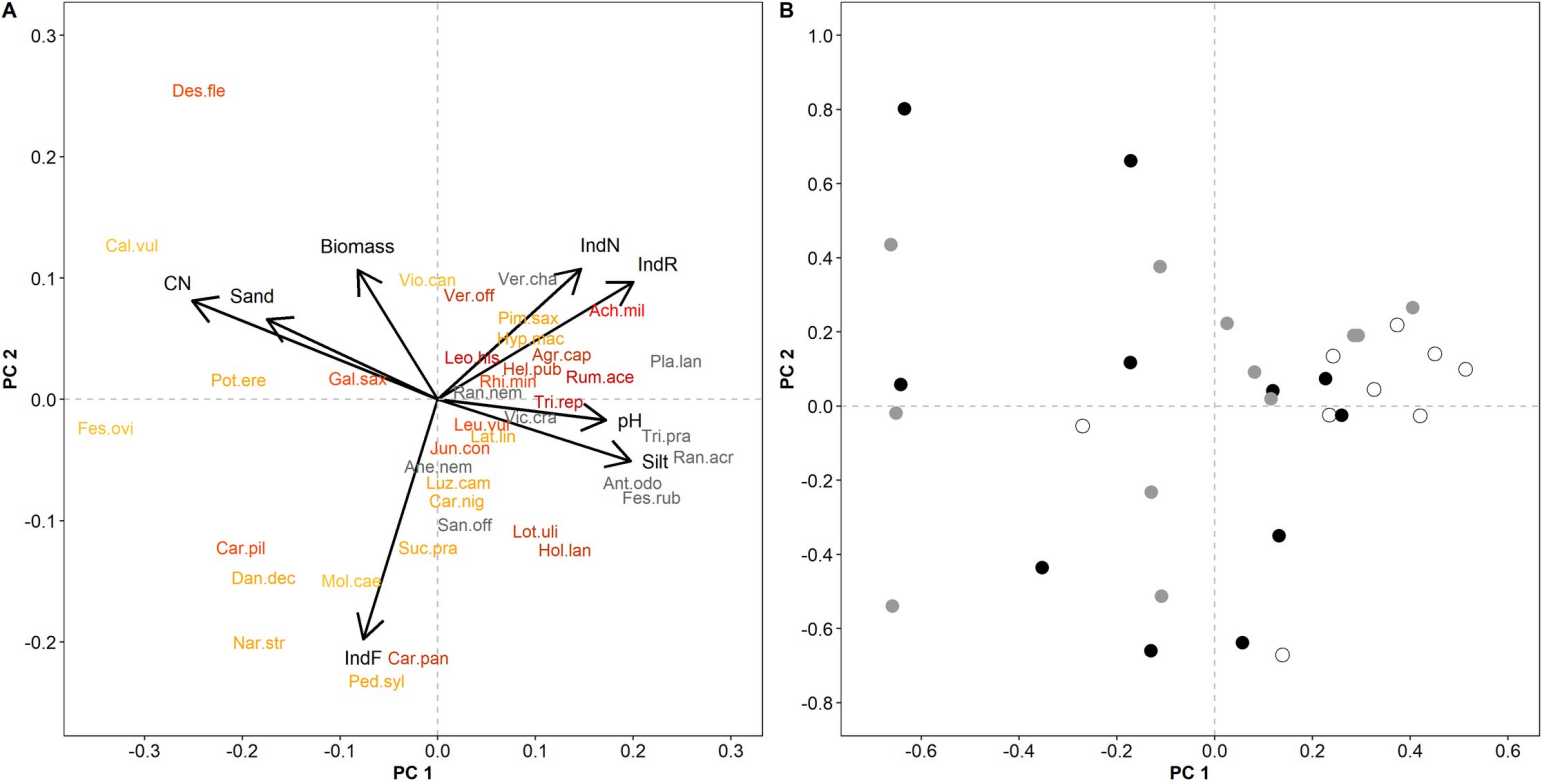
PCA results for plant species composition. (A) Species-environment biplot. First two components explained 33.3% of variance (22.5%, 10.8%). Abiotic and biotic variables that were significantly related to the PCA axes (p < 0.05, based on a permutation test with 10,000 permutations) were included post-hoc by projection. For clarity, only the 40 most frequent species are shown. Species names are colored according to their Ellenberg indicator N values from shades of red (N values of 5–6) to orange (3–4) and yellow (1–2) hues. For gray colored species names, no assigned indicator values were available. Abbreviations of parameter names are used according to Fig 2. (B) Scatter diagram with sample scores for the extinct (white), small (gray), and large (black) population sites.

Vegetation analysis revealed 112 vascular plant species on 32 sites with species numbers ranging from 12 to 42 species per site. The most common species were *Agrostis capillaris*, *Festuca rubra*, *Luzula campestris* and *Potentilla erecta*. Principal component analysis on species data (Fig 4A) revealed a similar picture as the PCA on environmental variables. The first PCA axis was negatively correlated with sand content, soil C:N ratio, and positively related to silt content and pH while the second axis was associated negatively with indicator F. Regarding the pattern of sites, extinct *A*. *montana* populations formed a relatively close group while small and large populations lay in a wide range along both axes (Fig 4B).

We colored the species names in Fig 4A according to their indicator N value and by comparing Fig 4A and 4B, a distinct clustering of species of nutrient rich habitats along with the extinct population sites can be observed.

Univariate analyses confirmed the relationships between environmental and vegetation site properties and population size categories as indicated by the results of the PCA. Indicator R value displayed a pattern similar to the pH values with extinct population sites revealing higher indicator R values than sites with existing populations (Fig 3C, F_2,29_ = 4.99, p = 0.014). Mean indicator N value was significantly lower on sites with existing populations than on extinct sites (Fig 3D, F_2,29_ = 9.87, p < 0.001). Distribution of herbs and grasses, moss, and litter on the overall biomass of 1 m^2^, as well as the relative amount of bare soil, did not differ among the categories (S2 Table). We did not find contrasting results comparing nutrients in the surrounding biomass and *A*. *montana* leaves among the three categories (S2 Table). However, several main factors were strongly associated with each other (S2 Fig). For example, C:N ratio was negatively correlated with pH, indicator R and indicator N values as well as species number, while pH and indicator N values were positively correlated. Sand content was strongly positively correlated with the overall biomass amount on 1 m^2^, cover of moss, and C:N ratio while it was negatively correlated with pH, indicator R value, and vascular plant biomass.

### Greenhouse experiment

**Fig 5:**
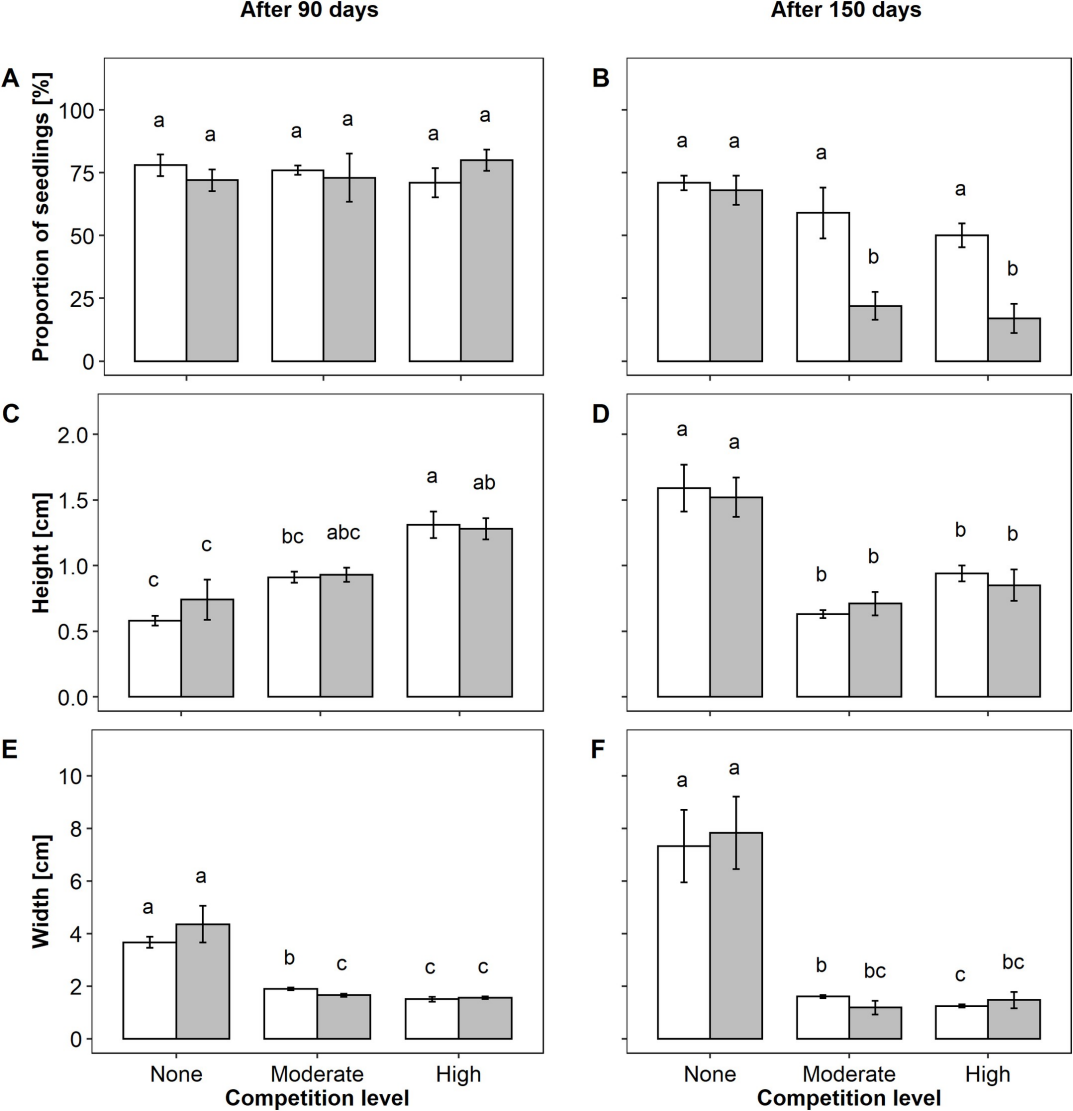
Response of *A*. *montana* seedlings to competition and N fertilization. Mean proportion of seedlings (A, B), height (C, D) and width (E, F) of rosettes for the competition levels (none, moderate, high) and the N fertilization levels (without fertilizer (white), with fertilizer (gray)) (± SE). Fig 5A, 5C, and 5E display the results after 90 days following sowing, whereas B, D, and F show the measurements after 150 days. Different letters indicate significant differences between treatment combinations; separately for the two sampling points (p < 0.05).

The proportion of *A*. *montana* seedlings was high in all treatments after 90 days (Fig 5A). At this time, the proportion of seedlings did not differ between treatments while significant differences in height and width could be observed (Table 1). Seedlings´ height was higher in plots without competition compared to the moderate and high competition level while the opposite was true for width (Fig 5C and 5E). 60 days later, i.e. 150 days after the sowing, the proportion of seedlings had declined along with increasing competition. While the unfertilized trays displayed only a slight decrease from no to high competition, fertilization led to a substantial drop in the proportion of seedlings (Fig 5B). ANOVA results also detected an interaction of the two factors (Table 1). Furthermore, rosettes were significantly higher and broader in the vegetation free plots compared to the competition levels after 150 days (Fig 5D and 5F). Here, the factor competition was shown to be the main cause for the differences (Table 1).

**Table 1:** Effects of treatments (competition, fertilization) and their interactions on *A*. *montana* seedlings. Shown are the results of analysis of variance on proportion of seedlings, height and width of rosettes concerning the two sampling points (90 and 150 days after seeding of *A*. *montana*) with degrees of freedoms (df), test statistics (F), and p-values.

Factors	df	Proportion of seedlings		Height of seedlings		Width of seedlings	
		F	p	F	p	F	p
**After 90 days**							
Competition	2	0.02	0.98	26.81	< 0.001	40.66	< 0.001
Fertilization	1	0.00	1	0.53	0.47	0.45	0.51
Comp:Fert	2	1.03	0.37	0.67	0.52	1.22	0.31
Residuals	24						
**After 150 days**							
Competition	2	18.68	< 0.001	31.57	< 0.001	38.65	< 0.001
Fertilization	1	22.77	< 0.001	0.08	0.78	0.03	0.87
Comp:Fert	2	4.43	0.02	0.34	0.72	0.18	0.84
Residuals	24						

## Discussion

Results from both field study and greenhouse experiment lead to consistent and coherent conclusions regarding the threats to *A*. *montana* in Hesse. Low soil C:N ratio and high indicator N on sites with extinct populations indicate a distinct nutrient enrichment of soils and indicate a strong relationship between this enrichment and the decline of *A*. *montana*. The species has an indicator N value as low as 2 [4] and Maurice et al. [9] found mean indicator N values for *A*. *montana* grasslands of 2.9. The values of the Hessian sites, especially those where *A*. *montana* had already become extinct, were much higher, reflecting an alarmingly high nutrient level of these habitats.

In general, a low soil sand content contributes to the nutrient enrichment as sand has a low capacity for exchanging and retaining nutrients and water [41], thus leading to a decreased N availability for plants in sandy soils. This is not supported explicitly by our field data as we did not find a relationship between sand content and Ellenberg indicator N value. However, positive, significant correlations between sand and *A*. *montana* rosette numbers as well as between sand and soil C:N indicate that sand content might play an important role. High sand content supports a low vegetation cover even under conditions of high atmospheric N deposition or nutrient leaching from surrounding agricultural fields. Consequently, this low vegetation cover provides more suitable habitat conditions for the competitively weak *A*. *montana*. However, sandy soils are known to have a diminished buffer capacity and might be more vulnerable to acidity [42]. In the Netherlands, studies have implied that N deposition itself is not the direct cause of shrinking *A*. *montana* populations but the following soil acidity [8]. However, the sampled sites in the Netherlands all had soil pH values of 3.7 and lower while the Hessian sites generally had higher values with an average of 4.1. In Hesse, we found significantly enhanced pH values on sites where *A*. *montana* became extinct which was underlined by higher R indicator values of the surrounding vegetation, highlighting the other side of the species´ tolerance range compared to the study by Fennema [8]. Taking into account that low pH values in soils are accompanied by a reduction of biological soil activity, and therefore the availability of nutrients [43, 44], we can assume that the observed higher pH values on sites of extinct *A*. *montana* populations contribute to the observed nutrient pattern. Several studies also found soil acidity to be of less importance, especially in the light of N deposition and its implications for competitive balance [14, 42]. A recent investigation also found an increase in pH and R indicator values over the last decades in acidic grasslands of the Werra-Meissner region, Central Germany, by resurveying permanent plots set up in the 1980s in 2012 [45]. The authors hypothesize that a reduction of airborne, acidifying compounds due to declining sulfur deposition rates since the 1990s could be a driver for species shifts in these grasslands.

With changing nutrient conditions, competitive relations are altered [42, 46] as plant species show particular reactions in answer to nutrient enrichment. For example, Barker et al. [18] found that invasion by *Deschampsia flexuosa* seedlings into a *Calluna* dominated heathland increased with N addition. Likewise, our greenhouse experiment showed that *A*. *montana* is inferior when growing in grass-dominated vegetation, especially with fertilization. Interestingly, the crucial stage was not germination but seedling establishment and growth. As an adaptation to competitive pressure, i.e. in order to increase light consumption, seedlings in the high-competition treatments were initially larger and more slender than those growing without accompanying vegetation. These adjustments are also prominent in *A*. *montana* plants on abandoned grasslands and other overgrown, uncultivated sites [10]. Later on in the greenhouse experiment, many seedlings died and the survivors showed a significant reduction in growth in comparison to the well-established rosettes of the individuals grown on bare soil. This is in line with findings by Loydi et al. [47] who found that negative competitive effects occur later during plant establishment while germination and early seedling establishment are not lowered by competition. We can conclude that both the field study and the greenhouse experiment, identified increasing competition through eutrophication as the main threat to *A*. *montana*.

Many studies identify matted layers of grass and moss as one of the main threats to endangered forbs [48–50]. Moss cover can indeed inhibit the establishment of seedlings, both when germinating beneath, or on top of the moss [51]. It may diminish light availability but can also protect seeds and seedlings against heavy frosts, high temperatures, and herbivores [51–53]. We were not able to determine moss cover as a crucial threat to *A*. *montana*. On the contrary, moss cover and biomass increased slightly with more rosettes and sand content. On sandy soils, vascular plant biomass, nutrients and therefore also competition, were lower, providing more suitable sites for *A*. *montana* plants and the positive effect on seedlings and grown plants might have compensated any negative impact by unfavorable moss growth.

The high amounts of nitrogen in the soil can be partly attributed to fertilization in the past. Still, nowadays many of the current *A*. *montana* populations are known to lie in established nature reserves, so that only leaching of nutrients from surrounding agricultural fields might be contributing to the increasing N content here. However, atmospheric N deposition due to emissions from combustion and agriculture has been intensifying [54]. Bobbink et al. [55] declared critical loads between 10–20 kg N ha^-1^ yr^-1^ for heath and grasslands. According to measurements by the German Environment Agency [56], values for N deposition on exemplary *A*. *montana* sites in Hesse lay between 15–21 kg N ha^-1^ yr^-1^ in 2004. The intensity of land use by grazing and mowing that is currently employed in many nature conservation areas seems to be too low to withdraw excess N sufficiently from the soils. The resulting dense vegetation, with or without considerable litter layers, might be able to host more or less stable *A*. *montana* populations but does not provide the necessities for regeneration and propagation. The aim remains to keep and create habitats with relatively open structures and low levels of nutrient supply for intact and regenerating *A*. *montana* populations.

In the current greenhouse experiment, seeds were in direct contact with the soil, which is often not the case for *A*. *montana* seeds under field conditions with thick layers of moss and litter preventing them to reach the ground. This might hinder seeds from germinating successfully or lead to fatal germination, i.e. seeds germinate but die before reaching the soil surface. Large seeded species like *A*. *montana* are especially prone to the latter when high amounts of litter prevent soil contact [51]. Consequently, as is the case for many other species, successful germination and establishment of *A montana* depends on gaps in the vegetation and presence of patches of bare ground that provide safe sites for plant regeneration [57]. Our field study did not reveal differences in bare soil cover along with the population size categories, but this might have been due to the small sampling area of only 1 m^2^ per site. Still, the greenhouse experiment clearly showed that bare ground, since it is accompanied by a low level of competition, is essential for seedling establishment.

In the field, we only included sites below 500 m above sea level because these are the most endangered populations in Hesse. General reasons for the better shape of populations in the mountainous regions might be harsher conditions with shorter vegetation periods, lower temperatures, and long periods of snow cover. These environmental circumstances and factors demand explicit adaptations thus equipping adapted species like *A*. *montana* with competitive advantages [9, 58]. However, facing threats arising from climate change, mountainous regions might soon face problems comparable to the lowlands. Increasing N deposition in these regions will be highly problematic for *A*. *montana* and other species requiring nutrient poor environments when conditions become more suitable for competitive species. Therefore, solutions are urgently needed to both reduce unintentional N deposition in the remaining nutrient poor areas as well as to implement different types and intensities of land use. In addition, alternative methods to create bare ground patches (e.g. controlled burning, removing vegetation as well as litter layers) need to be evaluated thoroughly for safekeeping and enhancement of self-sustainable populations.

## Supporting information

S1 Fig Mean proportions of soil particles for the three population size categories. Shown are proportions of sand (white), silt (light gray) and clay (dark gray).
(TIF)

S2 Fig Correlation matrix of variables for all sampled sites. Correlation coefficients are denoted using a color gradient from red (-1) to blue (1). Coefficients larger than 0.3 are displayed and significant relationships indicated by asterisk symbols (* p < 0.05, ** p < 0.01, *** p < 0.001). Biomass, VascPlants, Moss and Litter represent the cut and weighted biomass estimates on 1 m^2^; bare soil the cover percentage on 1 m^2^. SpecNo is an abbreviation for the absolute species number per site. IndN, IndR and IndF stand for the Ellenberg indicator values for nitrogen, reaction, and moisture.
(TIF)

S1 Table Field data of the 32 sampled sites in Hesse, Germany, including topographic, soil, and nutrient characteristics. (PDF)

S2 Table Differences in habitat characteristics between *A*. *montana* population size categories. Displayed are the mean ± SE values for the parameters of extinct (no rosettes), small (1–400 rosettes) and large populations (more than 400 rosettes). Also shown are F and *χ*, respectively, type of variable transformation (if applicable), as well as corresponding p-values.
(PDF)

S3 Table Vegetation data of the 32 sampled sites in Hesse, Germany. Vascular plant species were counted on two plots of 1 m^2^. Plot recordings consisted of presence-absence data of species in each grid cell. For the analyses, the number of presences of each species in the grid cells per site was used (summing up to a maximum of 50).
(PDF)

S4 Table Data from the greenhouse experiment. Shown are height and width measurements after 90 and 150 days for all seedlings grouped by box as well as the assigned treatment levels for the corresponding box numbers.
(PDF)
