# Supplementary figures and images for: From nutrients to competition processes: Habitat specific threats to Arnica montana L. populations in Hesse, Germany
Source: PLoS One. 2020 May 29;15(5):e0233709. doi: 10.1371/journal.pone.0233709 (PMC7259784; doi:10.1371/journal.pone.0233709)

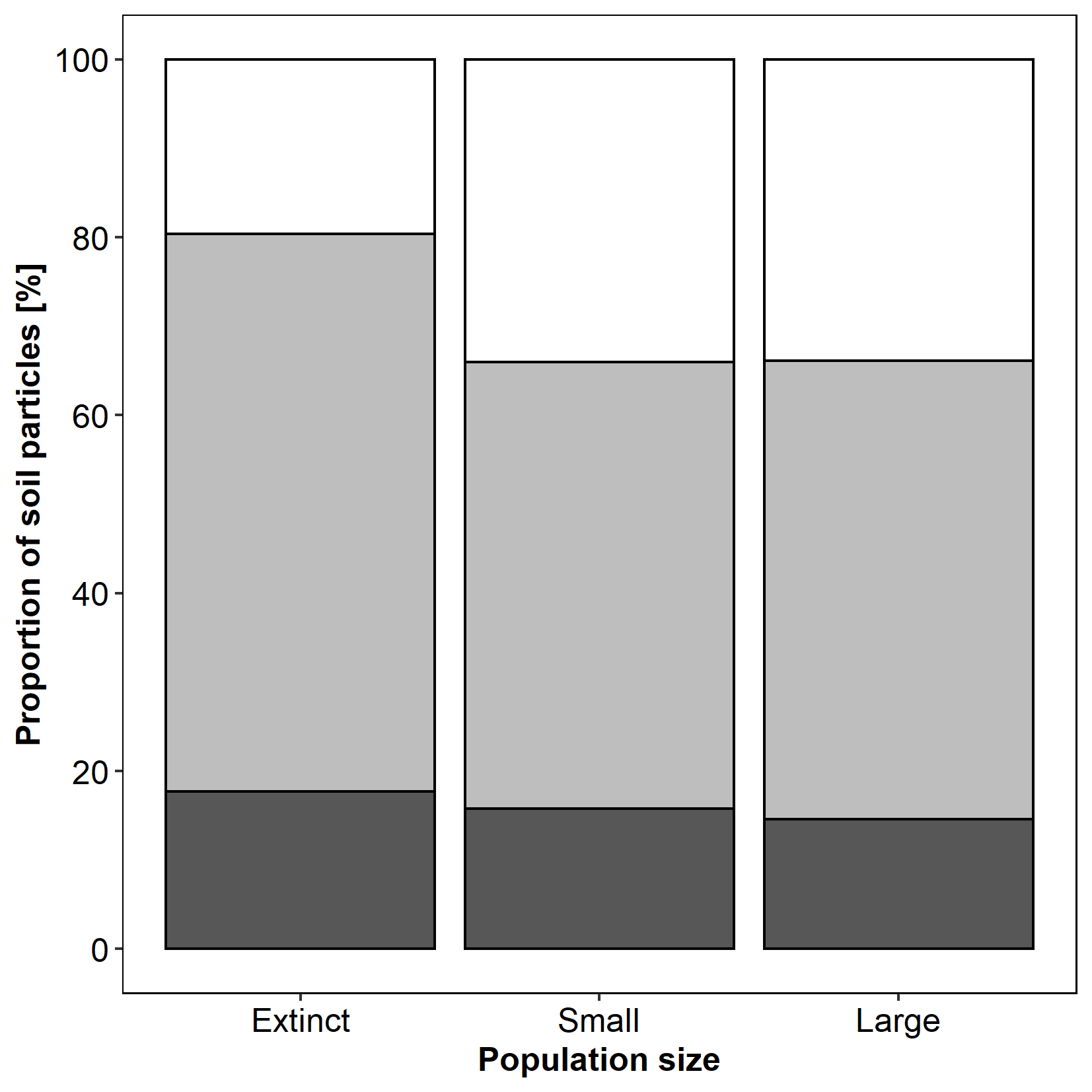

Supplement: S1 Fig — Shown are proportions of sand (white), silt (light gray) and clay (dark gray). (TIF) [file pone.0233709.s001.tif]

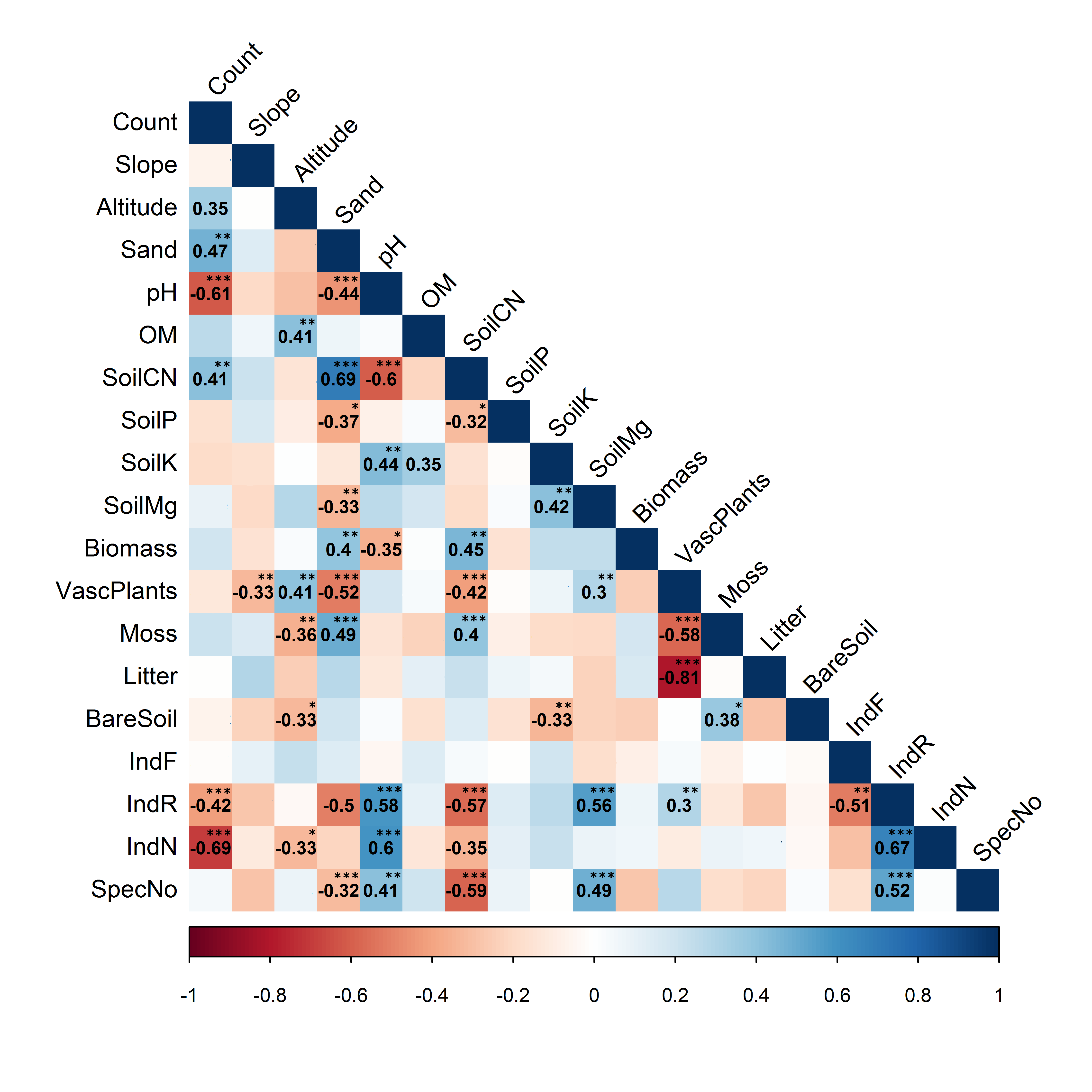

Supplement: S2 Fig — Correlation coefficients are denoted using a color gradient from red (-1) to blue (1). Coefficients larger than 0.3 are displayed and significant relationships indicated by asterisk symbols (* p < 0.05, ** p < 0.01, *** p < 0.001). Biomass, VascPlants, Moss and Litter represent the cut and weighted biomass estimates on 1 m2; bare soil the cover percentage on 1 m2. SpecNo is an abbreviation for the absolute species number per site. IndN, IndR and IndF stand for the Ellenberg indicator values for nitrogen, reaction, and moisture. (TIF) [file pone.0233709.s002.tif]
