# Supplementary material for: From nutrients to competition processes: Habitat specific threats to Arnica montana L. populations in Hesse, Germany
Source: PLoS One. 2020 May 29;15(5):e0233709. doi: 10.1371/journal.pone.0233709 (PMC7259784; doi:10.1371/journal.pone.0233709)
Supplement: S1 Table — (PDF) [file pone.0233709.s003.pdf]

Site	Population size	Pop. size category	Northness	Eastness	Slope [°]	Altitude [m.a.s.l.]	Sand content [%]	Silt content [%]	Clay content [%]
1	147	small	0.707	0.707	3.0	452	19.0	57.5	23.5
2	261	small	0.924	0.383	8.0	491	30.0	57.5	12.5
3	8875	large	0.707	0.707	7.0	262	51.0	45.0	4.0
4	443	large	1.000	0.000	6.0	390	19.0	57.5	23.5
5	7996	large	0.707	0.707	16.5	402	56.5	22.5	21.0
6	486	large	0.087	-0.996	5.5	363	9.0	74.0	21.0
7	0	extinct	-0.866	0.500	11.0	288	30.0	57.5	12.5
8	0	extinct	0.924	0.383	7.0	332	9.0	74.0	21.0
9	0	extinct	0.087	0.996	26.4	319	9.0	74.0	21.0
10	0	extinct	0.000	1.000	6.0	342	11.5	66.5	14.5
11	176	small	-0.707	0.707	5.0	384	30.0	57.5	12.5
12	0	extinct	0.000	1.000	7.0	308	34.0	45.0	21.0
13	0	extinct	-0.087	0.996	0.2	351	10.0	57.5	37.5
14	11	small	0.848	0.530	2.8	301	42.5	45.0	12.5
15	4300	large	0.000	1.000	32.5	527	19.0	57.5	23.5
16	1500	large	-0.906	-0.423	14.1	365	42.5	45.0	12.5
17	88	small	0.000	1.000	8.0	389	19.0	57.5	23.5
18	1615	large	0.383	0.924	13.5	392	51.0	45.0	4.0
19	100	small	-0.342	0.940	8.3	396	30.0	57.5	12.5
20	826	large	0.707	0.707	2.0	329	9.0	74.0	21.0
21	311	small	1.000	0.000	16.0	281	63.5	32.5	4.0
22	247	small	0.574	0.819	10.0	336	44.0	35.0	21.0
23	114	small	-0.643	0.766	4.1	507	20.0	40.0	40.0
24	6498	large	-0.996	0.087	5.0	439	51.0	45.0	4.0
25	0	extinct	0.707	0.707	6.0	480	30.0	57.5	12.5
26	409	large	-0.707	-0.707	4.0	361	44.0	35.0	21.0
27	184	small	0.819	-0.574	11.3	366	9.0	74.0	21.0
28	153	small	0.924	0.383	10.0	287	51.0	45.0	4.0
29	192	small	0.996	-0.087	13.4	286	51.0	45.0	4.0
30	0	extinct	1.000	0.000	3.0	464	38.7	55.9	5.5
31	0	extinct	-0.195	0.981	4.0	432	6.1	78.1	15.8
32	2157	large	-0.707	-0.707	2.0	472	23.6	69.0	7.3

Site	pH	Soil C content [%]	Soil N content [%]	Soil P [mg/kg]	Soil K [mg/kg]	Soil Mg [mg/kg]	Vascular plants [g/m²]	Moss [g/m²]	Litter [g/m²]	Bare soil [%]	Species number
1	3.9	57.2	4.8	15.0	96.3	6.0	333.10	21.90	285.20	3.5	31
2	4.2	64.8	5.8	13.4	58.3	6.0	388.40	48.00	233.50	1.0	31
3	4.2	52.7	3.7	3.8	43.4	3.0	65.90	68.40	105.20	8.0	31
4	4.1	57.3	4.8	12.2	99.1	18.0	512.00	95.80	432.20	3.5	42
5	3.8	39.3	2.4	6.8	129.0	13.0	696.10	784.40	580.50	2.5	18
6	4.1	38.7	3.5	13.8	120.0	7.0	389.70	94.90	135.20	0.0	23
7	4.4	47.5	4.4	17.0	99.7	6.0	426.20	19.80	281.10	1.0	26
8	4.7	53.0	5.1	16.4	80.5	11.0	436.10	111.30	364.50	1.0	28
9	4.7	40.9	3.7	22.6	171.0	7.0	261.20	116.80	273.80	0.0	27
10	4.5	46.1	3.9	15.6	87.9	7.0	288.70	97.80	93.50	10.0	25
11	4.0	52.2	4.2	16.2	82.8	5.0	NA	NA	NA	0.0	13
12	4.8	43.5	3.9	13.8	142.0	9.0	297.80	76.80	349.50	2.0	27
13	4.4	40.1	3.6	11.8	78.9	7.0	175.50	169.90	459.90	0.0	29
14	3.8	28.8	1.8	13.0	25.1	1.0	73.40	116.00	194.20	9.0	12
15	3.8	57.3	5.1	9.4	43.3	12.0	385.20	58.30	83.90	3.0	37
16	4.2	41.3	2.5	6.2	115.0	12.0	183.20	61.40	263.20	0.0	23
17	3.9	43.7	3.9	18.4	84.7	5.0	295.40	7.70	137.20	2.0	19
18	3.5	44.6	3.1	9.0	70.2	3.0	158.20	148.60	970.10	0.0	18
19	4.1	52.2	4.5	9.4	52.5	4.0	283.10	132.90	381.30	0.0	25
20	4.4	35.5	3.2	23.4	58.1	8.0	207.40	71.90	242.70	1.0	31
21	3.3	35.7	1.9	9.8	57.3	3.0	615.90	301.00	810.80	1.5	12
22	3.7	38.6	3.1	28.6	41.7	4.0	102.10	172.10	247.30	2.0	19
23	3.8	37.9	2.6	6.0	54.9	5.0	338.20	61.40	137.40	0.0	15
24	3.9	113.7	8.9	21.8	151.0	7.0	241.10	171.50	481.90	0.0	23
25	4.6	51.9	4.7	15.2	83.9	13.0	NA	NA	NA	6.5	31
26	4.1	34.7	2.9	10.4	48.9	6.0	249.10	178.10	203.20	2.0	39
27	3.6	40.3	3.2	50.6	47.9	9.0	295.50	98.00	306.00	0.0	31
28	4.0	34.6	2.7	17.8	46.3	3.0	333.70	186.20	220.50	9.0	29
29	4.3	47.1	3.6	8.4	79.8	6.0	247.10	134.50	213.40	2.0	34
30	4.1	5.5	0.4	NA	NA	6.0	360.10	135.60	297.10	1.0	23
31	4.0	4.1	0.3	NA	NA	11.0	644.10	201.70	660.60	0.5	22
32	4.0	6.4	0.5	NA	NA	11.0	273.30	138.80	275.80	1.5	35

Site	Ellenberg indicator values			Nutrients in *Arnica montana* leaves								
	Moisture (F)	Reaction (R)	Nitrogen (N)	N [%]	P in [%]	K [%]	Ca [%]	Mg [%]	Fe [ppm]	Zn [ppm]	Mn [ppm]	Cu [ppm]
1	6.087	3.462	2.958	1.5	0.1	2.7	1.1	0.6	72.7	96.5	576.4	14.9
2	5.095	4.615	3.727	1.4	0.1	0.8	1.5	0.9	205.7	101.0	353.1	12.2
3	4.810	3.471	3.087	1.1	0.1	2.2	1.0	0.3	98.5	68.0	569.7	11.3
4	4.862	4.867	3.265	1.2	0.1	2.2	1.8	0.8	108.8	99.1	259.6	10.5
5	5.364	4.400	3.500	0.9	0.1	2.8	1.1	0.5	75.1	53.2	371.6	13.8
6	5.333	3.667	3.125	NA	NA	NA	NA	NA	NA	NA	NA	NA
7	5.667	4.000	3.316	NA	NA	NA	NA	NA	NA	NA	NA	NA
8	5.143	5.167	4.263	NA	NA	NA	NA	NA	NA	NA	NA	NA
9	5.389	4.727	3.952	NA	NA	NA	NA	NA	NA	NA	NA	NA
10	5.933	4.889	3.765	NA	NA	NA	NA	NA	NA	NA	NA	NA
11	5.429	3.833	3.364	1.4	0.2	2.2	1.8	0.5	326.8	210.9	756.6	12.7
12	6.333	4.333	4.056	NA	NA	NA	NA	NA	NA	NA	NA	NA
13	5.250	5.000	3.850	NA	NA	NA	NA	NA	NA	NA	NA	NA
14	5.333	3.000	3.444	NA	NA	NA	NA	NA	NA	NA	NA	NA
15	5.348	4.667	3.071	NA	NA	NA	NA	NA	NA	NA	NA	NA
16	6.000	3.182	3.176	1.5	0.1	3.0	1.1	0.5	76.0	67.2	268.4	15.5
17	5.462	4.143	3.600	1.5	0.2	1.3	1.1	0.5	77.4	87.6	944.0	13.9
18	6.000	2.857	3.214	1.4	0.1	2.8	0.7	0.4	91.8	115.7	569.0	15.9
19	5.278	4.000	3.600	1.4	0.1	1.4	1.6	0.5	137.9	102.5	258.0	22.1
20	5.478	4.125	3.174	1.0	0.1	1.3	1.8	0.9	97.8	69.8	422.6	16.0
21	5.143	3.429	3.300	1.2	0.1	2.9	0.9	0.7	95.7	114.2	649.6	13.8
22	5.455	3.500	3.643	1.7	0.3	2.9	1.2	0.9	77.2	64.5	587.4	16.3
23	6.727	2.500	2.846	0.9	0.1	2.6	1.0	0.6	152.1	101.0	671.3	11.8
24	6.625	3.400	3.056	1.1	0.1	2.2	0.8	0.6	82.3	80.9	155.8	11.2
25	5.875	4.563	4.600	NA	NA	NA	NA	NA	NA	NA	NA	NA
26	6.370	3.556	3.310	1.1	0.1	1.2	2.0	1.3	111.5	84.6	748.0	10.1
27	5.391	4.133	3.292	1.5	0.1	1.2	1.1	0.7	86.9	135.0	307.4	15.1
28	6.400	3.333	3.261	1.3	0.1	3.3	0.7	0.6	132.5	253.0	1,186.0	19.7
29	5.174	4.467	3.577	0.8	0.1	2.4	1.5	0.6	152.7	115.5	178.7	19.5
30	6.765	3.786	3.063	NA	NA	NA	NA	NA	NA	NA	NA	NA
31	5.929	3.929	3.524	NA	NA	NA	NA	NA	NA	NA	NA	NA
32	6.042	3.941	3.320	1.2	0.1	1.2	1.4	1.5	78.6	114.2	289.2	11.3

Site	Nutrients in surrounding biomass								
	N [%]	P in [%]	K [%]	Ca [%]	Mg [%]	Fe [ppm]	Zn [ppm]	Mn [ppm]	Cu [ppm]
1	1.4	0.1	1.3	0.4	0.2	75.6	35.6	544.4	13.0
2	1.3	0.1	1.2	0.4	0.2	84.1	39.0	556.3	15.1
3	1.5	0.1	1.5	0.5	0.2	188.3	48.3	351.4	12.5
4	1.2	0.1	1.3	0.6	0.2	244.1	50.8	441.1	13.1
5	0.9	0.1	0.5	0.4	0.1	452.5	29.4	978.5	15.8
6	1.2	0.1	1.1	0.5	0.2	140.6	45.3	458.8	10.9
7	1.2	0.1	1.6	0.5	0.2	124.4	45.5	248.3	13.8
8	1.1	0.1	1.0	0.8	0.2	80.5	34.2	198.3	14.8
9	1.5	0.2	1.5	0.6	0.2	431.1	28.4	82.9	14.8
10	1.4	0.1	1.4	0.7	0.2	225.4	51.8	514.2	11.5
11	1.2	0.1	1.0	0.4	0.1	222.1	34.6	579.5	11.9
12	1.6	0.1	1.6	0.7	0.4	90.2	64.2	379.8	12.4
13	1.2	0.1	1.3	0.4	0.2	130.7	51.5	506.5	12.3
14	1.6	0.1	1.5	0.5	0.3	163.0	33.8	260.0	12.6
15	2.3	0.2	2.7	1.2	0.4	388.1	40.5	148.3	17.7
16	1.2	0.1	1.0	0.3	0.1	232.1	35.8	730.4	16.4
17	1.7	0.2	1.6	0.6	0.2	153.1	44.6	792.4	14.0
18	1.2	0.1	1.0	0.4	0.2	202.9	60.9	1,122.5	13.6
19	1.2	0.1	1.2	0.4	0.1	77.9	32.0	317.3	11.9
20	1.2	0.1	1.0	0.4	0.1	156.1	38.4	709.6	16.5
21	1.1	0.1	0.7	0.5	0.1	78.2	22.5	1,905.3	11.3
22	1.6	0.2	1.2	0.6	0.2	225.2	42.4	808.1	11.0
23	1.2	0.1	0.8	0.3	0.1	174.2	62.9	518.1	13.2
24	1.5	0.1	0.9	0.5	0.2	327.6	46.9	283.0	16.0
25	1.4	0.1	1.0	0.5	0.2	343.7	41.6	485.8	14.4
26	1.4	0.1	0.8	0.5	0.2	105.7	45.3	737.3	12.6
27	1.4	0.1	0.7	0.3	0.1	132.1	38.5	650.7	11.8
28	1.2	0.1	1.4	0.3	0.2	297.0	53.7	973.1	14.9
29	1.0	0.1	1.2	0.6	0.1	80.8	46.6	426.1	12.2
30	1.3	0.1	0.9	0.4	0.2	101.8	42.0	675.5	13.5
31	1.7	0.1	1.1	0.8	0.4	136.4	55.6	515.1	12.1
32	1.3	0.1	0.7	0.4	0.2	118.7	38.7	559.9	15.0
