# Supplementary material for: From nutrients to competition processes: Habitat specific threats to Arnica montana L. populations in Hesse, Germany
Source: PLoS One. 2020 May 29;15(5):e0233709. doi: 10.1371/journal.pone.0233709 (PMC7259784; doi:10.1371/journal.pone.0233709)
Supplement: S2 Table — Displayed are the mean ± SE values for the parameters of extinct (no rosettes), small (1–400 rosettes) and large populations (more than 400 rosettes). Also shown are F and χ, respectively, type of variable transformation (if applicable), as well as corresponding p-values. (PDF) [file pone.0233709.s004.pdf]

Parameter	Extinct populations	Small populations	Large populations	Statistics F/χ^2	Variable transformed	p
**Site characteristics**						
Slope	8 ± 3	8 ± 1	10 ± 3	4.36 (χ^2)	-	0.11
Northness	0.17 ± 0.17	0.42 ± 0.19	0.02 ± 0.21	1.04 (F)	-	0.37
Eastness	0.73 ± 0.11	0.46 ± 0.14	0.12 ± 0.22	3.07 (F)	-	0.06
Altitude [m.a.s.l.]	368 ± 24	373 ± 23	391 ± 21	0.27 (F)	-	0.76
**Soil measurements**						
Sand content [%]	19.8 ± 4.3	34.1 ± 4.7	34.1 ± 5.5	4.36 (χ^2)	-	0.11
Silt content [%]	62.9 ± 3.6	50.3 ± 3.4	51.8 ± 4.9	2.59 · (F)	squared	0.09
Clay content [%]	17.9 ± 3.0	15.9 ± 3.0	14.8 ± 2.6	0.28 (F)	-	0.76
pH	4.5 ± 0.1	3.9 ± 0.1	4.0 ± 0.1	13.32 (F)	-	< 0.001
C content [%]	37.0 ± 6.3	44.4 ± 3.0	47.4 ± 7.9	4.36 (χ^2)	-	0.11
N content [%]	3.3 ± 0.6	3.5 ± 0.4	3.7 ± 0.6	0.11 (F)	-	0.90
Soil C:N ratio	11.5 ± 0.3	13.2 ± 0.7	13.1 ± 0.6	7.24 (χ^2)	-	0.027
Organic matter content [%]	6.4 ± 1.1	7.7 ± 0.5	8.2 ± 1.4	0.77 (F)	-	0.47
Soil P content [mg/kg]	16.1 ± 1.1	17.2 ± 3.5	11.7 ± 1.9	1.92 (F)	log(x+1)	0.17
Soil K content [mg/kg]	106.3 ± 12.0	60.6 ± 6.0	87.8 ± 12.0	4.80 (F)	-	0.017
Soil Mg content [mg/kg]	8.6 ± 0.9	4.8 ± 0.6	9.1 ± 1.4	6.05 (F)	-	0.006
**Vegetation-rel. measurements**						
Vascular plant biomass [g/m²]	361.2 ± 50.6	300.5 ± 43.2	305.6 ± 54.0	0.24 (F)	-	0.78
Moss [g/m²]	116.2 ± 19.7	116.3 ± 25.5	170.2 ± 62.8	0.57 (F)	-	0.57
Litter [g/m²]	347.5 ± 57.9	287.9 ± 56.5	343.1 ± 78.9	0.03 (F)	-	0.97
Bare soil [%]	2.4 ± 1.1	2.5 ± 0.9	2.0 ± 0.7	0.06 (χ^2)	-	0.97
Number of species	26 ± 1	23 ± 2	29 ± 3	2.26 (F)	-	0.12
Shannon-Index	2.83 ± 0.05	2.63 ± 0.11	2.88 ± 0.12	1.96 (F)	log(x+1)	0.16
Evenness	0.87 ± 0.00	0.86 ± 0.01	0.86 ± 0.01	0.03 (F)	-	0.97
Mean N indicator value	3.82 ± 0.16	3.38 ± 0.08	3.21 ± 0.04	11.09 (χ^2)	-	0.004
Mean F indicator value	5.81 ± 0.17	5.58 ± 0.15	5.66 ± 0.18	0.45 (F)	-	0.64
Mean R indicator value	4.49 ± 0.17	3.70 ± 0.18	3.83 ± 0.19	4.99 (F)	-	0.014
**Nutrients in surrounding biomass**						
N [%]	1.38 ± 0.06	1.33 ± 0.06	1.36 ± 0.11	0.10 (F)	-	0.90
P [%]	0.13 ± 0.01	0.11 ± 0.01	0.11 ± 0.01	0.31 (χ^2)	-	0.86
K [%]	1.26 ± 0.09	1.15 ± 0.09	1.12 ± 0.17	2.41 (F)	-	0.30
Ca [%]	0.58 ± 0.05	0.44 ± 0.03	0.52 ± 0.08	5.14 (χ^2)	-	0.08
Mg [%]	0.24 ± 0.03	0.16 ± 0.01	0.19 ± 0.02	2.23 (F)	squared	0.12
Fe [ppm]	184.9 ± 41.4	146.9 ± 21.0	232.4 ± 34.1	2.37 (F)	log(x+1)	0.11
Zn [ppm]	46.1 ± 3.7	40.5 ± 3.1	43.7 ± 2.5	0.81 (F)	-	0.45
Mn [ppm]	400.7 ± 63.0	694.3 ± 124.8	592.8 ± 89.3	2.46 (F)	log(x+1)	0.10
Cu [ppm]	13.3 ± 0.4	12.8 ± 0.4	14.6 ± 0.6	3.65 (F)	-	0.039
**Nutrients in A. montana leaves**						
N [%]	N/A	1.32 ± 0.08	1.15 ± 0.06	1.57 (F)	-	0.13
P [%]	N/A	0.14 ± 0.02	0.11 ± 0.01	63 (χ^2)	-	0.11
K [%]	N/A	2.16 ± 0.24	2.10 ± 0.22	0.16 (F)	-	0.88
Ca [%]	N/A	1.22 ± 0.10	1.31 ± 0.14	0.39 (F)	-	0.70
Mg [%]	N/A	0.64 ± 0.04	0.76 ± 0.12	0.76 (F)	-	0.46
Fe [ppm]	N/A	138.0 ± 21.7	91.1 ± 4.2	67 (χ^2)	-	0.20
Zn [ppm]	N/A	125.6 ± 16.2	86.7 ± 6.6	2.49 (F)	log(x+1)	0.023
Mn [ppm]	N/A	588.0 ± 88.2	406.0 ± 57.3	1.63 (F)	-	0.12
Cu [ppm]	N/A	15.63 ± 1.03	12.84 ± 0.81	74.5 (χ^2)	-	0.063
