# Supplementary material for: From nutrients to competition processes: Habitat specific threats to Arnica montana L. populations in Hesse, Germany
Source: PLoS One. 2020 May 29;15(5):e0233709. doi: 10.1371/journal.pone.0233709 (PMC7259784; doi:10.1371/journal.pone.0233709)
Supplement: S3 Table — Vascular plant species were counted on two plots of 1 m2. Plot recordings consisted of presence-absence data of species in each grid cell. For the analyses, the number of presences of each species in the grid cells per site was used (summing up to a maximum of 50). (PDF) [file pone.0233709.s005.pdf]

Species / Site	1	2	3	4	5	6	7	8	9	10	11	12	13	14	15	16	17	18	19	20	21	22	23	24	25	26	27	28	29	30	31	32
Achillea millefolium	0	0	20	6	0	27	19	0	8	0	0	21	45	0	7	0	24	0	50	6	0	0	0	0	10	0	25	5	0	0	0	0
Achillea ptarmica	0	0	0	0	0	0	8	0	0	1	0	16	0	0	1	0	0	0	0	0	0	0	0	0	0	0	0	0	0	0	0	0
Agrostis canina	0	0	0	0	0	0	0	0	0	0	0	0	0	0	0	0	0	0	0	0	0	0	0	0	0	0	0	0	0	5	0	0
Agrostis capillaris	50	44	50	50	1	0	39	50	49	50	50	50	50	25	48	17	50	50	50	49	0	37	0	26	50	19	50	34	49	31	50	17
Ajuga reptans	0	0	0	0	0	0	0	0	0	0	0	0	0	0	0	0	0	0	0	18	0	0	0	0	3	29	0	1	0	3	5	18
Alchemilla vulgaris	0	0	0	5	0	0	0	0	0	0	0	0	0	0	0	0	0	0	0	0	0	0	0	0	2	0	0	0	0	0	0	0
Anemone nemorosa	2	41	0	0	0	0	0	0	0	0	0	0	0	0	0	0	0	0	38	33	5	19	0	18	0	13	38	18	25	0	0	16
Anthoxanthum odoratum	0	5	5	11	0	37	49	35	14	16	1	4	24	0	48	1	5	0	18	16	0	21	0	11	32	18	10	8	8	43	0	13
Arnica montana	0	0	16	0	20	0	0	0	0	0	0	0	0	0	0	3	0	6	0	1	0	0	0	2	0	0	0	0	0	0	0	3
Arrhenaterum elatius	0	8	0	0	0	0	0	1	0	0	0	0	0	0	0	0	0	0	0	0	0	0	0	0	0	0	0	0	0	0	0	0
Bellis perennis	0	0	0	0	0	0	0	0	0	0	0	0	0	0	0	0	0	0	0	0	0	0	0	0	12	0	0	0	0	0	0	0
Betonica officinalis	0	9	0	26	0	0	4	0	0	0	0	0	0	0	0	0	0	0	0	0	0	0	0	0	0	1	0	0	0	0	0	0
Betula pendula juv.	11	0	8	0	1	0	0	0	0	0	0	0	0	0	0	0	0	2	0	0	0	0	0	0	0	0	0	0	0	0	0	0
Bistorta officinalis	0	0	0	0	0	0	0	0	0	0	0	0	0	0	0	0	0	0	0	0	0	0	3	46	0	0	0	0	0	0	2	0
Briza media	0	1	0	10	0	27	1	0	0	0	0	0	0	0	1	0	0	0	0	0	0	0	0	0	0	0	0	0	15	0	0	0
Calluna vulgaris	0	0	8	0	25	0	0	0	0	0	0	0	0	22	0	29	0	0	0	0	20	0	14	0	0	0	0	0	0	0	4	0
Campanula rotundifolia	2	5	0	0	0	0	0	0	1	0	11	0	7	0	1	0	1	0	5	0	0	4	0	0	0	0	0	0	3	0	0	0
Cardamine pratensis	0	0	0	0	0	0	0	0	0	0	0	0	0	0	0	0	0	0	0	0	0	0	0	0	3	1	0	0	0	0	0	0
Carex caryophyllea	0	0	0	0	0	0	0	0	12	0	0	0	0	0	0	0	0	0	0	0	0	0	0	0	0	0	0	0	47	0	0	0
Carex hirta	0	0	0	0	0	0	0	0	0	0	0	17	0	0	0	0	0	0	0	0	0	0	0	0	0	0	0	0	0	0	0	0
Carex leporina	0	0	0	0	0	0	0	0	0	0	0	0	0	0	0	0	0	0	0	0	0	0	0	0	6	2	0	4	0	1	0	0
Carex muricata	0	0	0	0	0	0	0	0	0	0	0	0	0	0	0	0	0	0	0	0	0	0	0	0	0	1	0	0	0	0	0	4
Carex nigra	0	0	0	0	0	0	0	0	0	0	0	7	0	0	0	0	0	0	0	0	0	0	0	0	8	44	0	18	0	0	0	6
Carex pallescens	0	0	0	0	0	0	0	0	0	2	0	0	0	0	0	0	0	0	0	36	0	0	0	0	0	0	0	12	0	0	0	0
Carex panicea	4	0	0	0	0	0	0	0	0	0	0	0	0	0	0	0	0	0	0	4	0	0	3	20	0	23	0	25	0	34	0	47
Carex pilulifera	4	0	0	30	0	0	0	0	0	0	0	0	0	6	29	24	0	3	2	13	7	2	21	29	0	16	1	4	0	1	18	29
Carpinus betulus	0	0	0	0	0	0	0	0	0	0	0	0	0	0	0	1	0	4	0	0	0	1	0	0	0	0	0	23	0	0	0	1
Centaurea jacea	0	1	0	11	0	0	0	0	11	3	0	0	11	0	4	0	0	0	0	0	0	0	0	0	0	2	0	0	20	0	0	3
Centaurea nigra	0	0	0	0	0	0	0	0	0	0	0	0	0	0	0	0	0	0	0	0	0	0	0	0	0	0	20	0	0	0	0	0
Cerastium holosteoides	0	0	0	0	0	0	0	0	0	0	0	0	1	0	0	0	0	5	0	0	0	0	0	0	10	0	0	0	0	0	0	0
Cirsium palustre	6	0	0	0	0	0	0	0	0	0	0	3	0	0	0	1	0	0	0	0	0	0	0	0	0	4	0	0	0	5	0	0
Cynosurus cristatus	0	0	0	0	0	0	0	7	0	0	0	0	0	0	0	0	0	0	0	0	0	0	0	0	4	0	0	0	0	0	0	0

**Species / Site**	**1**	**2**	**3**	**4**	**5**	**6**	**7**	**8**	**9**	**10**	**11**	**12**	**13**	**14**	**15**	**16**	**17**	**18**	**19**	**20**	**21**	**22**	**23**	**24**	**25**	**26**	**27**	**28**	**29**	**30**	**31**	**32**
Dactylis glomerata	0	0	0	1	0	0	0	0	0	0	0	0	0	0	2	0	0	2	0	0	0	0	0	0	0	0	0	0	0	0	1	0
Danthonia decumbens	4	0	0	31	0	0	2	0	0	8	0	0	0	21	21	27	0	0	0	19	1	0	21	22	0	21	4	7	19	0	3	31
Deschampsia cespitosa	17	0	0	0	0	1	0	0	0	0	0	11	0	0	0	0	0	0	0	0	0	0	0	3	2	0	0	0	0	0	6	0
Deschampsia flexuosa	0	0	35	0	46	0	0	0	0	0	0	0	0	1	0	8	0	0	0	0	31	24	0	0	0	0	0	0	1	0	0	0
Epilobium angustifolium	0	0	0	0	0	0	0	0	0	0	0	0	0	0	0	0	0	0	0	0	4	0	0	0	0	0	0	0	0	0	0	0
Euphrasia officinalis	0	0	0	0	0	0	0	0	0	0	0	0	0	0	0	0	0	0	0	0	0	0	0	0	0	6	0	0	0	0	0	0
Festuca ovina	4	0	24	3	12	0	0	0	0	0	0	0	0	45	0	2	0	4	0	0	6	8	48	49	0	13	0	1	19	0	40	32
Festuca pratensis	0	3	0	0	0	0	0	0	0	0	0	0	0	0	0	0	0	0	0	0	0	0	0	0	1	0	0	0	0	0	0	0
Festuca rubra	50	50	1	50	1	50	50	50	50	50	50	50	50	0	50	2	50	41	50	42	0	39	8	15	17	50	50	50	48	50	25	33
Galium saxatile	11	0	0	0	0	0	0	0	0	0	25	4	0	0	0	23	22	50	20	0	0	44	0	50	0	0	18	8	0	0	0	4
Galium verum	0	17	3	1	0	0	0	0	0	0	0	0	0	0	0	0	0	0	0	0	0	0	0	0	0	0	0	0	0	0	0	0
Genista germanica	0	0	22	0	12	0	5	0	0	0	0	0	0	0	0	0	0	0	0	0	0	0	0	0	0	0	0	0	0	0	0	0
Genista sagittalis	0	0	21	0	0	0	0	0	0	0	0	0	0	0	0	0	0	0	0	0	0	0	0	0	0	0	0	0	0	0	0	0
Genista tinctoria	0	0	0	16	0	0	0	0	0	0	0	0	0	0	0	0	0	0	0	0	0	0	0	0	0	0	0	0	0	0	0	0
Helianthemum nummulariur	0	0	0	15	0	0	0	0	0	0	0	0	0	0	0	0	0	0	0	0	0	0	0	0	0	0	0	0	0	0	0	0
Helictotrichon pratense	0	0	0	22	0	0	0	0	0	0	0	0	0	0	8	0	0	0	0	0	0	1	0	0	0	0	0	0	0	0	0	0
Helictotrichon pubescens	0	23	1	14	0	3	4	24	13	0	0	4	2	0	0	0	0	0	0	0	0	0	0	1	0	5	0	0	1	0	0	0
Hieracium spec.	11	0	0	14	8	38	27	0	6	30	2	11	15	3	1	3	0	1	0	0	0	37	5	11	0	0	0	13	39	0	7	0
Holcus lanatus	1	2	0	5	0	39	23	25	6	0	4	0	0	0	2	0	3	0	3	24	0	0	0	0	15	12	1	27	9	32	1	18
Holcus mollis	0	0	0	0	0	0	0	0	0	0	0	0	0	0	0	0	50	24	0	0	0	0	0	0	0	0	0	0	0	0	0	0
Hypericum maculatum	3	26	0	0	0	0	0	20	0	0	0	0	42	0	0	0	40	0	45	0	0	0	0	0	0	0	0	0	0	0	36	0
Hypericum perforatum	0	0	27	0	0	1	0	0	0	0	0	0	0	0	0	0	0	0	0	0	0	0	0	0	0	0	0	0	0	0	0	0
Hypochaeris radicata	0	0	0	0	0	0	0	0	7	12	0	0	0	0	0	0	0	0	0	0	0	0	0	0	0	0	0	0	0	0	0	0
Juncus acutiflorus	0	0	0	0	0	0	0	0	0	0	0	0	0	0	0	0	0	0	0	0	0	0	0	0	0	16	0	50	0	17	0	0
Juncus conglomeratus	0	0	0	0	0	0	0	0	0	1	0	1	0	0	0	0	0	0	0	7	0	0	0	0	3	0	0	6	0	0	0	0
Juncus effusus	0	0	0	0	0	0	0	0	0	0	0	0	0	0	0	0	0	0	0	0	0	0	0	0	0	0	0	0	0	0	0	0
Knautia arvensis	0	7	0	1	0	0	0	2	19	0	0	0	16	0	1	0	0	0	0	0	0	0	0	0	0	0	0	0	4	0	0	0
Lathyrus linifolius	0	17	0	0	0	0	0	0	0	0	0	0	0	0	25	0	0	0	26	50	0	5	0	0	0	17	36	0	0	0	3	0
Lathyrus pratensis	0	0	0	0	0	0	0	0	3	0	0	6	3	0	0	0	0	0	0	0	0	0	0	0	0	0	0	0	0	0	0	0
Leontodon autumnalis	0	0	0	0	0	0	0	0	0	0	0	0	0	0	0	0	0	0	16	0	0	0	0	0	32	0	1	0	17	0	0	0
Leontodon hispidus	0	6	0	8	0	0	0	0	0	0	0	0	0	0	44	0	0	0	0	0	0	0	0	0	0	0	0	0	43	0	0	0
Leucanthemum vulgare	0	0	0	15	0	13	0	0	0	15	0	0	4	0	0	0	0	0	0	14	0	0	0	0	0	0	5	0	14	0	0	0

Species / Site	1	2	3	4	5	6	7	8	9	10	11	12	13	14	15	16	17	18	19	20	21	22	23	24	25	26	27	28	29	30	31	32
Lotus corniculatus	0	0	0	31	0	0	0	6	0	19	0	0	0	0	0	0	0	0	0	0	0	0	0	0	0	0	0	0	1	0	0	0
Lotus uliginosus	30	0	0	0	0	20	0	0	37	0	0	0	46	0	2	0	43	0	15	21	0	0	0	15	2	9	2	0	4	25	4	38
Luzula campestris	41	20	35	39	2	41	47	9	49	40	50	1	46	8	42	1	45	20	50	48	20	5	16	50	28	42	47	43	31	35	37	42
Lychnis flos-cuculi	0	0	0	0	0	0	0	0	0	0	0	0	0	0	0	0	0	0	0	0	0	0	0	0	0	1	0	0	0	3	0	0
Melampyrum sylvaticum	0	0	0	0	0	0	0	0	0	0	0	0	0	0	0	0	0	0	0	0	0	15	0	0	0	0	0	0	0	0	0	0
Molinia caerulea	18	0	0	0	0	0	0	0	0	0	0	0	0	0	0	1	0	0	0	0	0	0	20	11	0	12	0	12	0	0	0	32
Nardus stricta	3	0	0	8	0	4	3	0	0	0	0	0	0	22	5	20	0	7	0	43	0	0	50	8	0	12	5	3	5	17	6	34
Pedicularis sylvatica	3	0	0	0	0	0	0	0	0	0	0	0	0	0	0	0	0	0	0	0	0	0	31	3	0	36	0	20	0	49	0	21
Phyteuma nigrum	0	13	0	0	0	0	0	5	0	0	0	0	0	0	0	0	0	0	0	0	0	0	0	0	0	0	0	0	0	0	0	0
Pimpinella saxifraga	1	8	2	33	1	8	0	5	11	0	0	0	8	0	36	0	3	0	10	7	0	0	0	0	0	0	4	0	24	0	0	0
Pinus sylvestris	0	0	1	0	3	0	0	0	0	0	0	0	0	10	0	0	0	0	0	0	0	0	0	0	0	0	0	0	0	0	0	0
Plantago lanceolata	0	36	23	45	0	23	21	48	36	19	0	8	22	0	48	0	18	0	3	11	0	0	0	2	30	13	12	0	44	1	0	30
Platanthera chlorantha	2	4	0	0	0	0	0	1	0	0	0	0	0	0	1	0	0	0	0	0	0	0	0	0	0	0	0	0	0	0	0	0
Poa pratensis	0	0	0	0	0	0	1	4	4	1	0	3	0	0	0	0	4	0	0	0	0	0	0	0	0	0	0	0	0	0	0	0
Polygala vulgaris	0	0	7	15	0	0	0	0	0	0	0	0	1	0	9	1	0	0	1	1	0	0	0	0	0	7	0	0	12	0	0	7
Populus tremula	5	0	1	0	0	0	0	0	0	0	0	0	0	0	0	2	0	0	0	0	0	0	0	0	0	0	0	0	0	0	0	0
Potentilla erecta	48	6	45	30	11	44	34	0	0	38	47	37	27	2	46	33	16	40	18	43	35	2	32	41	0	25	44	48	48	2	50	50
Prunella vulgaris	0	0	0	0	0	0	0	0	0	0	0	0	0	0	1	0	0	0	0	27	0	0	0	0	25	0	0	0	0	0	0	13
Pyrus pyraster juv.	0	0	0	0	0	0	0	0	0	0	0	0	0	0	0	0	0	0	0	0	12	0	0	0	0	0	0	0	0	0	0	0
Ranunculus acris	0	27	0	3	0	0	18	44	14	36	0	21	29	0	23	0	19	0	17	38	0	0	0	0	25	40	0	0	0	24	0	4
Ranunculus flammula	0	0	0	0	0	0	0	0	0	0	0	0	0	0	0	0	0	0	0	0	0	0	0	0	0	0	0	0	0	0	0	0
Ranunculus nemorosus	0	0	0	0	0	41	35	0	0	0	0	0	0	0	0	0	0	0	0	0	0	0	0	0	0	0	24	0	16	0	0	0
Ranunculus polyanthemos	3	7	0	33	0	0	0	0	0	0	18	0	0	0	0	0	0	0	0	0	0	0	0	0	0	0	0	0	0	0	0	0
Rhinanthus minor	0	0	47	15	0	29	3	41	0	1	0	0	0	0	48	0	0	0	0	0	0	0	0	0	0	31	25	2	0	0	0	7
Rumex acetosa	9	25	4	1	0	6	29	40	7	9	11	10	14	0	26	0	1	18	7	12	0	1	0	0	25	1	1	2	4	0	14	7
Sagina apetala	0	0	0	0	0	0	0	0	0	0	0	0	0	0	0	0	0	0	0	0	0	0	0	0	13	0	0	0	0	0	0	0
Sanguisorba minor	0	0	0	35	0	0	0	0	0	0	0	0	0	0	0	0	0	0	0	0	0	0	0	0	0	0	0	0	0	0	0	0
Sanguisorba officinalis	0	0	0	7	0	5	0	20	0	3	0	10	1	0	0	0	0	0	0	0	0	0	1	50	1	21	6	9	0	6	0	0
Selinum carvifolia	0	0	0	0	0	0	13	0	0	0	0	0	0	0	0	0	0	0	0	3	0	0	0	0	0	0	0	0	0	0	0	0
Solidago virgaurea	0	0	0	0	21	0	0	0	0	0	0	0	0	0	0	0	0	0	0	0	0	0	0	0	0	0	1	0	0	0	0	0
Stellaria graminea	0	0	1	0	0	0	0	17	16	0	0	3	25	0	2	0	0	0	0	0	0	0	0	0	0	0	0	0	0	0	0	0
Succisa pratensis	40	0	0	0	0	0	0	0	0	36	0	0	0	0	0	0	0	0	0	12	0	0	1	4	0	0	4	6	1	7	7	20

Species / Site	1	2	3	4	5	6	7	8	9	10	11	12	13	14	15	16	17	18	19	20	21	22	23	24	25	26	27	28	29	30	31	32
Taraxacum officinale	0	8	0	12	0	0	3	8	3	7	0	0	0	0	0	0	7	0	2	0	0	0	0	0	6	0	0	0	0	0	0	0
Teesladia nudicaulis	0	0	12	0	0	0	0	0	0	0	0	0	0	0	0	0	0	0	0	0	0	0	0	0	0	0	0	0	0	0	0	0
Trifolium alpestre	0	0	0	0	0	0	0	0	0	0	0	0	0	0	0	0	0	0	0	0	0	0	0	0	0	0	0	0	0	0	22	0
Trifolium dubium	0	0	0	0	0	0	0	0	0	6	0	0	0	0	0	0	0	0	0	0	0	0	0	0	2	3	0	0	10	0	0	0
Trifolium medium	0	0	0	0	0	0	0	0	0	0	0	0	0	0	10	0	0	0	0	0	0	0	0	0	0	0	7	0	0	0	0	0
Trifolium pratense	0	30	0	29	0	50	47	20	48	31	0	0	0	0	41	0	0	0	0	27	0	0	0	0	3	33	0	0	33	0	0	8
Trifolium repens	0	0	0	0	0	0	0	0	0	43	9	19	22	0	0	0	0	0	25	0	0	0	0	0	47	0	14	0	0	0	0	32
Trisetum flavescens	0	0	0	0	0	0	0	1	4	0	0	0	23	0	3	0	0	0	6	0	0	0	0	0	0	0	0	0	0	0	0	0
Vaccinium myrtillus	0	0	0	0	0	0	0	0	0	0	0	0	0	0	0	40	0	14	0	0	50	0	0	0	0	0	0	0	0	0	0	0
Vaccinium vitis-idaea	0	0	0	0	0	0	0	0	0	0	0	0	0	0	0	0	0	0	0	0	38	0	0	0	0	0	0	0	0	0	0	0
Veronica chamaedrys	3	31	11	0	0	0	0	3	0	0	0	0	9	0	8	0	49	1	49	2	0	2	0	0	0	0	0	0	7	0	0	0
Veronica officinalis	5	21	18	0	0	0	0	0	25	0	50	0	0	0	0	0	0	0	0	5	0	41	0	0	0	0	3	0	5	0	0	7
Veronica serpyllifolia	0	0	0	0	0	0	0	0	0	0	0	0	0	0	0	0	0	0	0	0	0	0	0	0	8	0	0	0	0	0	0	0
Vicia cracca	0	0	0	9	0	7	16	13	0	0	0	19	35	0	0	0	0	0	0	0	0	0	0	0	0	26	3	0	0	0	0	1
Vicia sepium	0	0	0	0	0	0	0	0	9	0	0	0	0	0	0	0	0	0	0	0	0	0	0	0	0	0	0	0	0	0	0	0
Viola canina	0	0	35	45	4	0	1	0	0	0	0	0	0	0	46	0	0	0	0	0	0	0	0	0	0	0	7	0	0	0	0	0
