# Supplementary material for: From nutrients to competition processes: Habitat specific threats to Arnica montana L. populations in Hesse, Germany
Source: PLoS One. 2020 May 29;15(5):e0233709. doi: 10.1371/journal.pone.0233709 (PMC7259784; doi:10.1371/journal.pone.0233709)
Supplement: S4 Table — Shown are height and width measurements after 90 and 150 days for all seedlings grouped by box as well as the assigned treatment levels for the corresponding box numbers. (PDF) [file pone.0233709.s006.pdf]

Box number	Seedling number/box	1	2	3	4	5	6	7	8	9	10	11	12	13	14	15	16	17	18	19	20
1	Height [cm] - 90 days	0.7	0.6	0.7	0.5	0.9	0.6	0.7	0.6	0.9	0.4	0.7	0.7	0.5	0.4	0.8					
	Width [cm] - 90 days	5.7	1.7	2.4	5.7	5.9	4.8	4.7	7.7	4.9	3.2	6.4	0.3	2.9	3.5	5.4					
	Height [cm] - 150 days	2.1	1.6	2.3	1.9	3.2	1.8	1.8	1.7	2.5	2.0	2.8	2.0	2.3	1.6	2.3					
	Width [cm] - 150 days	6.8	8.8	14.5	16.0	13.5	7.0	9.2	13.0	10.8	8.8	14.0	12.3	11.3	8.3	10.6					
2	Height [cm] - 90 days	0.8	0.7	0.7	0.5	0.8	0.8	0.8	0.8	0.2	0.6	0.9	0.5	0.2	0.6	0.7	0.6	0.4	0.5		
	Width [cm] - 90 days	2.9	2.5	4.2	1.8	4.9	4.2	4.6	3.1	3.7	4.8	5.5	2.8	1.7	4.1	3.0	2.6	1.8	1.5		
	Height [cm] - 150 days	0.6	1.1	1.3	1.1	0.6	1.0	0.4	0.8	1.0	2.3	1.1	0.9	0.9	1.3						
	Width [cm] - 150 days	0.5	4.6	5.0	2.1	1.3	1.5	1.2	2.6	4.7	8.8	1.6	1.8	2.3	8.4						
3	Height [cm] - 90 days	0.4	1.1	0.6	0.9	0.7	0.6	1.0	0.7	0.6	0.7	0.4	0.9	0.3	0.6	0.5	0.2	0.2			
	Width [cm] - 90 days	3.2	4.6	3.7	6.4	3.8	2.4	9.0	4.0	3.8	3.7	4.3	2.3	1.9	3.5	5.2	2.0	2.0			
	Height [cm] - 150 days	2.0	2.3	1.5	0.8	1.3	0.9	0.9	1.0	1.6	1.5	1.6	1.4	2.4							
	Width [cm] - 150 days	11.0	16.0	4.0	1.4	3.5	3.5	1.4	2.4	5.9	8.6	8.5	4.1	11.7							
4	Height [cm] - 90 days	0.6	0.4	0.3	0.4	0.5	0.4	0.3	0.7	0.4	0.2	0.6	0.4	0.4							
	Width [cm] - 90 days	6.2	4.7	2.1	1.5	2.0	2.2	2.6	6.4	2.9	2.1	5.0	4.9	4.7							
	Height [cm] - 150 days	1.9	2.0	1.7	1.7	1.6	2.4	2.9	1.4	1.9	1.5	1.3	1.1	1.4							
	Width [cm] - 150 days	13.0	13.1	0.6	9.1	5.4	11.0	16.0	14.0	8.6	11.6	10.1	5.5	9.6							
5	Height [cm] - 90 days	0.8	0.2	0.6	0.5	1.0	0.6	0.5	0.6	0.5	0.5	0.4	0.5	0.6	0.6	0.7					
	Width [cm] - 90 days	5.2	2.0	5.6	1.8	4.5	3.7	2.3	1.4	3.4	4.0	2.2	1.6	2.2	2.7	4.5					
	Height [cm] - 150 days	1.5	1.7	1.5	1.6	2.0	1.2	1.2	1.4	1.1	1.6	1.3	1.6	2.3	1.6	1.6	1.9				
	Width [cm] - 150 days	4.8	6.4	4.5	6.7	6.3	3.4	3.2	7.8	3.7	5.0	6.7	5.4	9.1	6.8	7.4	11.2				
6	Height [cm] - 90 days	1.0	0.8	0.6	1.0	0.7	1.0	0.9	0.6	0.4	0.5	0.6	0.4								
	Width [cm] - 90 days	6.2	7.0	4.8	4.9	3.7	4.9	5.4	1.8	3.7	5.0	3.8	1.7								
	Height [cm] - 150 days	1.1	2.3	1.3	2.9	1.7	1.7	0.9													
	Width [cm] - 150 days	1.5	10.3	5.2	16.0	2.7	1.3	1.7	1.7	12.2	5.5										
7	Height [cm] - 90 days	1.5	0.6	0.7	0.7	0.1	1.0	0.5	0.7	0.5	0.5	1.0	0.6	0.6	0.9	1.0	0.8	1.1			
	Width [cm] - 90 days	11.5	7.7	6.0	5.5	1.7	5.7	7.0	3.7	6.7	2.6	7.5	2.9	5.3	4.4	3.4	4.8	8.2			
	Height [cm] - 150 days	0.9	1.1	1.2	1.7	1.4	1.6	1.2	0.9	2.1	0.9	1.2	1.1	1.3	1.5	0.9	1.6				
	Width [cm] - 150 days	7.9	12.4	6.5	10.3	7.9	9.5	2.4	2.3	3.4	1.2	8.5	1.7	2.5	14.8	11.9	4.6				

Box number	Seedling number/box	1	2	3	4	5	6	7	8	9	10	11	12	13	14	15	16	17	18	19	20
**8**	Height [cm] - 90 days	0.4	0.7	0.7	0.5	1.0	0.6	0.8	0.8	0.5	0.6	0.7	0.6	0.7	1.0	0.7	0.7				
	Width [cm] - 90 days	2.3	4.9	2.4	2.0	4.6	4.6	6.5	3.0	2.7	4.6	5.0	2.0	3.0	4.8	4.4	5.9				
	Height [cm] - 150 days	1.3	1.8	2.0	1.4	1.2	1.1	2.3	1.7	1.3	1.8	1.5	1.2	1.6	1.7	1.6	1.1				
	Width [cm] - 150 days	11.0	15.5	17.5	16.5	10.6	5.4	14.2	15.2	13.8	11.9	17.5	5.9	5.3	17.8	14.7	5.9				
**9**	Height [cm] - 90 days	0.9	1.3	0.7	0.6	5.6	0.7	0.7	0.9	0.9	1.0	1.0	1.0	1.0							
	Width [cm] - 90 days	6.0	7.7	6.7	5.0	6.2	6.3	3.4	4.0	7.5	6.5	4.5	6.5	6.4							
	Height [cm] - 150 days	1.5	1.9	3.1	1.9	3.4	1.7	2.0	1.9	1.6	1.3	1.7	1.3								
	Width [cm] - 150 days	11.7	7.4	10.9	10.8	10.0	8.6	12.4	14.5	7.2	6.2	5.3	7.8								
**10**	Height [cm] - 90 days	0.4	0.2	0.5	0.6	0.5	0.2	0.2	0.2	0.2	0.3	0.3	0.2	0.2	0.3	0.2					
	Width [cm] - 90 days	1.9	2.9	4.6	1.7	2.4	3.1	1.4	1.7	1.7	1.2	2.0	1.6	1.1	0.7	1.4					
	Height [cm] - 150 days	1.1	0.8	1.7	1.3	0.7	1.3	0.8	1.5	2.1	0.4	0.8	1.3	0.8	1.0						
	Width [cm] - 150 days	7.3	1.6	1.6	3.9	1.5	8.2	2.6	10.6	5.2	0.8	11.6	7.2	3.2	1.9						
**11**	Height [cm] - 90 days	1.0	1.6	0.6	0.7	0.3	1.2	1.5	1.0	0.7	0.7	0.8	1.1	0.7	0.7	1.1					
	Width [cm] - 90 days	1.8	2.3	1.7	1.8	1.3	2.4	2.3	2.5	2.5	1.7	2.5	1.9	2.4	1.6	1.9					
	Height [cm] - 150 days	0.8	0.6	0.5	0.8	0.7	0.6	0.8	0.6	0.6	0.8	0.7	0.9	0.6	0.6	0.9					
	Width [cm] - 150 days	2.2	1.9	1.5	1.7	1.9	1.7	1.9	1.9	1.8	2.2	1.4	1.4	1.7	1.7	1.6					
**12**	Height [cm] - 90 days	1.6	1.2	0.7	0.7	0.5	0.6	0.8	0.9	0.8	1.3	0.8	0.6	0.7	0.9						
	Width [cm] - 90 days	2.3	1.9	1.6	1.6	1.9	2.2	1.8	2.0	1.5	2.2	1.7	2.0	1.9	1.9						
	Height [cm] - 150 days	0.6	0.7	0.6	0.4	0.6	1.0	0.6	0.7	0.4	0.4	0.4	0.3								
	Width [cm] - 150 days	1.8	1.5	1.8	1.8	1.7	2.0	2.3	1.6	1.7	1.1	1.1	1.4								
**13**	Height [cm] - 90 days	0.9	0.5	0.8	0.9	1.0	1.1	1.2	1.0	1.0	1.3	1.0	0.9	1.1	1.1	1.2	1.0				
	Width [cm] - 90 days	2.4	1.8	1.9	1.8	1.9	2.1	2.0	1.8	2.0	1.9	1.6	1.8	2.1	2.3	2.0	1.8				
	Height [cm] - 150 days	0.6	0.9	0.8	0.6	0.7	0.5	0.6	0.4	1.0	0.5	0.5	0.6	0.6	0.7	0.5					
	Width [cm] - 150 days	1.7	1.2	2.6	1.8	1.3	1.9	1.9	0.6	1.5	2.2	0.8	0.8	1.9	1.6	0.7					
**14**	Height [cm] - 90 days	1.1	1.1	0.9	0.8	0.9	0.7	1.8	1.2	1.0	0.8	1.1	0.8	0.8	1.1	0.8					
	Width [cm] - 90 days	1.8	2.1	1.9	0.9	1.7	2.1	2.4	2.4	1.9	1.5	1.9	2.0	1.9	1.9	1.3					
	Height [cm] - 150 days	1.1	0.5	0.6	0.5																
	Width [cm] - 150 days	2.2	0.9	1.8	1.6																

Box number	Seedling number/box	1	2	3	4	5	6	7	8	9	10	11	12	13	14	15	16	17	18	19	20
**15**	Height [cm] - 90 days	0.6	0.8	0.6	1.2	1.7	1.0	1.0	0.5	0.6	0.8	0.6	0.2	0.8	0.5	0.9	0.6				
	Width [cm] - 90 days	1.6	1.6	1.9	1.8	2.1	1.4	2.0	1.5	1.3	2.0	1.4	1.4	2.0	1.6	1.9	1.8				
	Height [cm] - 150 days	0.4	0.4	0.7	0.5	0.6	0.8	0.5	0.3	0.5	0.9	0.8	0.5	0.6							
	Width [cm] - 150 days	1.5	1.1	1.5	1.9	1.1	0.8	1.6	1.3	2.1	1.7	1.5	1.3	1.4							
**16**	Height [cm] - 90 days	0.6	0.6	0.7	0.4	0.7	1.1	1.2	0.8	1.2											
	Width [cm] - 90 days	1.2	1.7	2.0	1.4	1.7	1.7	1.8	1.0	1.5											
	Height [cm] - 150 days	0.9	0.4	0.7	0.7	0.7	1.2	0.9	0.6												
	Width [cm] - 150 days	1.8	2.3	2.3	1.9	1.9	2.0	2.2	1.6												
**17**	Height [cm] - 90 days	1.2	0.9	1.4	1.2	0.8	1.0	1.2	1.1	0.4	1.4	0.9	1.1	1.9	1.0	1.0	1.1	1.4			
	Width [cm] - 90 days	1.9	2.0	2.2	2.0	1.5	1.5	2.4	2.1	0.9	2.1	2.4	2.4	2.6	1.6	1.7	1.8	1.4			
	Height [cm] - 150 days	0.3	0.7	1.2	0.4	1.0	0.6														
	Width [cm] - 150 days	1.1	0.6	2.9	1.2	2.4	1.2														
**18**	Height [cm] - 90 days	0.9	0.8	1.3	0.8	1.0	0.4	1.2	0.8	0.6	0.5	0.5	0.8	1.2	1.6	0.9	1.3	0.5			
	Width [cm] - 90 days	1.9	1.1	0.9	1.7	1.7	1.7	1.6	1.9	1.5	1.8	2.4	0.2	2.0	1.7	1.8	1.5	2.0			
	Height [cm] - 150 days	0.3	0.5	0.6																	
	Width [cm] - 150 days	0.3	1.2	1.1																	
**19**	Height [cm] - 90 days	0.8	0.6	1.3	0.5	1.0	1.3	0.7	1.0	0.9	0.9	1.1	1.4	1.0	0.9	0.4	0.8	0.3			
	Width [cm] - 90 days	0.9	0.7	1.6	1.3	1.3	1.8	2.1	1.9	2.0	1.9	2.1	1.9	1.5	1.5	1.9	1.4	1.6			
	Height [cm] - 150 days	0.6	0.6																		
	Width [cm] - 150 days	0.4	0.7																		
**20**	Height [cm] - 90 days	0.7	0.7	1.6	0.6	0.8	1.0	0.6	0.8	1.8	0.7	1.2	0.7	1.2	1.2	0.9	0.8				
	Width [cm] - 90 days	1.4	1.6	2.0	1.1	1.2	2.3	1.9	1.6	2.1	1.4	1.6	1.2	1.3	1.3	1.4	1.6				
	Height [cm] - 150 days	1.3	1.3	0.5																	
	Width [cm] - 150 days	0.3	1.9	0.5																	
**21**	Height [cm] - 90 days	1.6	1.0	1.3	0.8	1.1	1.2	0.7	1.0	1.4	0.5										
	Width [cm] - 90 days	1.9	2.0	1.0	1.4	1.7	1.3	1.6	1.2	1.7	0.8										
	Height [cm] - 150 days	1.4	0.5	1.1	1.2	1.1	0.9	1.2	1.2												
	Width [cm] - 150 days	1.4	1.0	1.1	0.9	1.3	0.7	1.0	2.3												

Box number	Seedling number/box	1	2	3	4	5	6	7	8	9	10	11	12	13	14	15	16	17	18	19	20
**22**	Height [cm] - 90 days	1.9	1.6	1.7	1.3	1.1	1.7	1.4	1.8	1.2	1.4	2.2	1.6	1.7	1.3	1.8	1.3	0.8	1.5		
	Width [cm] - 90 days	1.9	2.3	1.9	1.2	1.3	1.4	1.9	1.7	1.3	1.3	1.8	1.1	1.4	1.6	1.9	1.5	1.5	1.7		
	Height [cm] - 150 days	0.5	0.6	1.0	0.6	0.6	0.8	0.4	0.5	1.1	1.1	1.0	1.2	0.6							
	Width [cm] - 150 days	0.6	1.3	1.8	1.4	1.3	0.6	2.0	1.2	2.1	1.1	1.0	0.8	0.7							
**23**	Height [cm] - 90 days	1.6	1.6	1.7	1.3	1.3	1.8	1.8	1.3	1.7	2.3	1.4	1.2	1.9	1.2						
	Width [cm] - 90 days	1.9	1.6	1.7	1.0	0.9	1.7	1.7	1.6	1.2	1.4	2.3	1.9	1.7	1.9						
	Height [cm] - 150 days	0.8	0.7	1.4	1.1	1.0	0.9	1.0	1.1	0.9	0.9										
	Width [cm] - 150 days	1.4	1.9	0.3	1.0	1.3	1.8	1.2	1.3	1.4	1.4										
**24**	Height [cm] - 90 days	1.6	0.6	0.9	0.8	1.6	0.7	1.9	1.7	1.4	1.2	0.6	1.7	1.2	1.5						
	Width [cm] - 90 days	0.6	1.6	0.6	1.2	0.6	0.9	1.6	0.9	1.5	1.3	1.4	1.8	0.9	1.6						
	Height [cm] - 150 days	0.9	1.3	0.5	1.1	0.8	0.7	1.0	1.0	0.8	0.6	0.7									
	Width [cm] - 150 days	0.8	0.3	1.0	1.0	1.1	1.0	0.6	1.3	1.7	1.3	1.4									
**25**	Height [cm] - 90 days	1.1	1.2	1.1	0.7	1.5	1.5	0.5	1.4	1.4	1.2	1.6	1.6	0.6	0.7						
	Width [cm] - 90 days	2.2	1.1	2.1	1.0	1.2	1.7	1.1	2.1	1.8	1.6	2.0	1.9	1.6	2.1						
	Height [cm] - 150 days	1.3	1.3	0.7	0.9	1.2	1.0	1.1	1.0	0.6											
	Width [cm] - 150 days	1.8	1.5	2.0	0.9	1.3	1.1	0.8	1.0	2.2											
**26**	Height [cm] - 90 days	1.4	1.5	1.1	1.8	1.2	1.6	1.9	0.8	0.9	1.3	1.9	0.9	1.3	2.4	1.5	0.7				
	Width [cm] - 90 days	2.2	1.9	1.1	1.7	0.9	1.4	1.5	0.7	1.7	1.3	1.3	1.4	1.5	2.4	1.2	1.5				
	Height [cm] - 150 days	0.9	0.7	0.9																	
	Width [cm] - 150 days	1.4	1.3	0.9																	
**27**	Height [cm] - 90 days	2.0	1.1	1.9	1.3	1.5	1.7	1.1	1.7	1.3	1.2	1.7	1.7	1.3	1.8						
	Width [cm] - 90 days	2.0	1.7	1.6	1.4	1.5	1.8	1.7	1.1	1.6	0.8	1.3	1.5	1.8	0.8						
	Height [cm] - 150 days	0.9	0.6	1.3	1.3	1.0															
	Width [cm] - 150 days	1.2	1.2	1.1	2.6	1.4															
**28**	Height [cm] - 90 days	0.9	1.0	1.1	1.7	0.9	0.9	1.5	1.5	0.9	0.5	1.2	0.4	1.0	1.2	0.8	1.4	1.1			
	Width [cm] - 90 days	2.1	1.6	1.1	2.1	1.4	1.2	1.8	1.0	2.0	1.6	1.5	1.5	1.9	1.5	1.5	1.5	1.8			
	Height [cm] - 150 days	1.0																			
	Width [cm] - 150 days	2.4																			

Box number	Seedling number/box	1	2	3	4	5	6	7	8	9	10	11	12	13	14	15	16	17	18	19	20
29	Height [cm] - 90 days	1.2	0.2	1.2	0.9	0.8	1.7	1.9	1.0	0.8	1.2	2.3	1.2	1.2							
	Width [cm] - 90 days	1.7	1.4	1.9	1.7	1.7	2.0	1.9	1.6	1.0	1.4	2.2	1.8	2.1							
	Height [cm] - 150 days	0.4																			
	Width [cm] - 150 days	0.5																			
30	Height [cm] - 90 days	1.5	1.0	1.2	1.0	1.6	1.2	1.0	1.1	1.3	0.5	1.1	1.6	0.9	1.5	1.7	1.4	1.2	1.2		
	Width [cm] - 90 days	1.2	0.9	1.8	0.9	1.6	2.4	1.6	1.5	1.7	1.5	1.5	1.2	1.7	1.8	2.2	1.3	1.2	1.4		
	Height [cm] - 150 days	1.3	1.1	0.9	0.9	0.9	0.9	1.0													
	Width [cm] - 150 days	1.3	1.1	1.9	1.2	2.4	2.3	2.1													

Box number	Competition level	Fertilizer treatment
1	None	None
2	None	None
3	None	None
4	None	None
5	None	None
6	None	Plus nitrogen
7	None	Plus nitrogen
8	None	Plus nitrogen
9	None	Plus nitrogen
10	None	Plus nitrogen
11	Moderate	None
12	Moderate	None
13	Moderate	None
14	Moderate	None
15	Moderate	None

Box number	Competition level	Fertilizer treatment
16	Moderate	Plus nitrogen
17	Moderate	Plus nitrogen
18	Moderate	Plus nitrogen
19	Moderate	Plus nitrogen
20	Moderate	Plus nitrogen
21	High	None
22	High	None
23	High	None
24	High	None
25	High	None
26	High	Plus nitrogen
27	High	Plus nitrogen
28	High	Plus nitrogen
29	High	Plus nitrogen
30	High	Plus nitrogen
